# The embryonic development of the central American wandering spider *Cupiennius salei*

**DOI:** 10.1186/1742-9994-8-15

**Published:** 2011-06-14

**Authors:** Carsten Wolff, Maarten Hilbrant

**Affiliations:** 1 Humboldt-Universität zu Berlin Institut für Biologie/Vergleichende Zoologie Philippstraße 13, 10115 Berlin, Germany; 2 Universität zu Köln Institut für Genetik, Zülpicher Straße 47a, 50674 Köln, Germany; 3 Oxford Brookes University Headington Campus Gipsy Lane, Oxford OX3 0BP, UK

## Abstract

**Background:**

The spider *Cupiennius salei *(Keyserling 1877) has become an important study organism in evolutionary and developmental biology. However, the available staging system for its embryonic development is difficult to apply to modern studies, with strong bias towards the earliest developmental stages. Furthermore, important embryonic events are poorly understood. We address these problems, providing a new description of the embryonic development of *C. salei*. The paper also discusses various observations that will improve our understanding of spider development.

**Results:**

Conspicuous developmental events were used to define numbered stages 1 to 21. Stages 1 to 9 follow the existing staging system for the spider *Achaearanea tepidariorum*, and stages 10 to 21 provide a high-resolution description of later development. Live-embryo imaging shows cell movements during the earliest formation of embryonic tissue in *C. salei*. The imaging procedure also elucidates the encircling border between the cell-dense embryo hemisphere and the hemisphere with much lower cell density (a structure termed 'equator' in earlier studies). This border results from subsurface migration of primordial mesendodermal cells from their invagination site at the blastopore. Furthermore, our detailed successive sequence shows: 1) early differentiation of the precheliceral neuroectoderm; 2) the morphogenetic process of inversion and 3) initial invaginations of the opisthosomal epithelium for the respiratory system.

**Conclusions:**

Our improved staging system of development in *C. salei *development should be of considerable value to future comparative studies of animal development. A dense germ disc is not evident during development in *C. salei*, but we show that the gastrulation process is similar to that in spider species that do have a dense germ disc. In the opisthosoma, the order of appearance of precursor epithelial invaginations provides evidence for the non-homology of the tracheal and book lung respiratory systems.

## Background

The field of research aiming to clarify the evolution of development and the relationship between developmental changes and phenotypic evolution is called evolutionary developmental biology (or 'evo-devo'). A common evo-devo strategy is to compare the development of so-called model organisms, which are often chosen because of their phylogenetic position. For example, based on gene expression data in spider embryos, it has been proposed that the parasegmental boundary is a conserved trait in arthropod development [1]. The argument was that spiders are part of a basally branching clade (chelicerates) within the euarthropods [2,3] and that shared traits between spiders and any other arthropods are thus likely to reflect the arthropod ancestral state [1]. Another reason to select a particular model species is for its ability to give new insights into a specific evolutionary developmental theme [4], such as the evolution of novelties. The many evolutionary adaptations specific to spiders make them particularly suitable for this research theme. Examples include the tubular trachea; the silk producing system; complex silk spinning behaviour and the remarkable optical system. All these features probably contributed to the success of spiders in occupying a broad range of terrestrial and even aquatic ecosystems. These diverse spider adaptations studied in a phylogenetic context may shed more light on their evolution as well as on the principles of adaptive evolution in general.

It is therefore very promising that two spider species, *Cupiennius salei *and *Achaearanea tepidariorum *have emerged over the years as experimental models for embryological studies [5]. *C. salei*, a wandering spider (Ctenidae), has been the subject of neurological, physiological and behavioural studies over many decades [e.g. [6-8]]. More recently, this species has also been used for evo-devo studies [e.g. [9-15]]. It is especially suitable for developmental studies because of easy maintenance and the high number of large eggs available throughout the year. Other benefits include the possibility of performing functional analyses of genes via embryonic RNAi, in-situ hybridisation in advanced developmental stages and the relative ease of dissection of its large embryos [16,17]. *A. tepidariorum*, a cobweb spider (Theridiidae) has also become very popular for evo-devo studies in recent years [5]. The advantages of this species include a short generation time, parental RNAi and in-situ hybridisation of the earliest stages. These features make *A. tepidariorum *a particularly suitable subject for the study of early development. Its ecology also differs significantly from that of *C. salei*. For example, *A. tepidariorum *captures prey by using silk [18,19], whereas *C. salei *does not use its silk for this purpose. The silk spinning organs, which relate to this difference in behaviour, differ in their morphology as well [5,20] and differentiate during late embryonic development. The two species thus complement one another as laboratory model organisms for the comparison of chelicerate embryology with those of other major arthropod taxa. These species also exhibit organ differences that may have been crucial in their evolution. Furthermore, molecular techniques available for both species permit detailed comparisons between them.

In order to facilitate cross-species comparisons in evo-devo studies, clear and comparable embryological staging systems are required. Such systems have proved indispensible for the study of other arthropods such as the insect *Drosophila melanogaster *[21,22] or the crustaceans *Parhyale hawaiensis *[23] and *Porcellio scaber *[24]. Unfortunately, comparably clear and comprehensive systems are not currently available for spider development. For *A. tepidariorum*, the early stages of development (until about the first appearance of the prosomal appendages) have recently been well defined [25]. Later embryonic and post-embryonic stages have not been defined for this species. A more complete staging system for *C. salei *does exist, covering the whole of its embryonic development [26] but this suffers from several flaws. First, this system is strongly biased towards early development and misses important aspects of late ontogeny. Second, it is based on embryonic timing (measured in hours after egg laying: hAEL). Timing is inappropriate for *C. salei *because of variations in the developmental rate of the eggs of different broods. Third, the images and drawings featured in that staging system are not detailed enough for comparison with modern imaging methods such as confocal scans [e.g. [27]]. This makes it difficult to draw comparisons between recent studies on organogenesis in *C. salei*, including those on heart development [28], brain development [27] and limb development [29]. It is even more difficult to draw comparisons between *C. salei *and other spider species.

A new morphological description of *C. salei *development is presented in this paper. Detailed pictures based on live-embryo imaging, scanning electron microscopy (SEM) and fluorescent staining are also presented. In addition we look critically at the existing terminology for the first post-embryonic stages. The result is a series of 21 discrete embryonic stages that are linked cohesively to the first post-embryonic stages. All the stages can be easily identified via examination of living animals and by the use of common fluorescent markers on fixed specimens, thus providing a practical basis for future evo-devo studies.

Our observations have also allowed us to add new data to some long-standing morphological debates. One such problem area relates to gastrulation. Gastrulation is the morphogenetic process that separates an initially simple sheet of cells (the blastoderm) into the germ layers (ectoderm, mesoderm and endoderm) thereby reorganizing the tissue with a greater degree of complexity. In spiders, gastrulation starts with the internalization of cells at the blastopore (or 'gastral groove' [30]). Gradually, a primary thickening (or primitive plate) is formed around the area of the blastopore [26,31]. With multiple cell layers underneath the blastoderm (now ectoderm) this area represents the mesendodermal mass (mesodermal and endodermal cells). In many spider species, the formation and subsequent differentiation of the blastopore takes place at the centre of a structure called a germ disc [31,32]. This is a regional differentiation of blastodermic cells that migrate together, aggregating in the form of a disc. Such germ discs, as seen for example in *A. tepidariorum*, exhibit a high cell density in relation to the extra-embryonic part of the egg [e.g. [5]]. The rim of the germ disc appears as a border circling the whole egg.

In *C. salei*, a dense germ disc is not formed [5,26]. In this species, before gastrulation the blastodermic cells in the embryonic portion of the egg do not appear to be denser than those of the extra-embryonic portion. Nevertheless, towards the end of gastrulation a visible border circling the whole egg appears between the embryonic and extra-embryonic regions. This border has been referred to in the past as the 'equator' [26]. The nature of the equator and the reason for this difference between *C. salei *and those species with a dense germ disc are poorly understood. In this publication, with the first live-embryo imaging data for *C. salei*, we offer an explanation for the appearance of the equator and compare our findings with conditions in species that do exhibit a dense germ disc.

Another complex aspect of spider embryonic development is the differentiation of the anterior-most part of the germ band. This part of the germ band consists of a precheliceral region, a cheliceral segment and a pedipalpal segment. The precheliceral region is composed of two large precheliceral lobes, with the anlage of the stomodeum (mouth opening) in between. These precheliceral lobes subdivide and later give rise to the protocerebrum, including the optic lobes. This subdivision comes about via the folding and packing of distinct areas into cerebral grooves (semi-lunar grooves) or vesicles.

Some putative relationships between the cerebral grooves and various other brain structures have been suggested for a number of spider species [33-37] and other arachnids [38,39]. For instance a recent SEM study on the grey widow spider *Latrodectus geometricus*, deals with the development of the precheliceral region [37] but the processes of migration and overgrowing of single brain compartments are not clearly documented. Another recent study on the development of the protocerebrum in *C. salei *demonstrated the major morphogenetic movements involved in the formation of the brain centres (including the optic centre, mushroom body and arcuate body). This study also included the expression of several genes that might play a role in this process [27]. However, the early developmental sequence of these movements is not described in fine detail.

In the present investigation, we show in detail the sequence of early differentiation of what will eventually become the individual brain parts from the precheliceral neuroectoderm of *C. salei*. This serves to complement and extend earlier studies of brain development in this species [27]. We show which areas of the precheliceral lobe the brain components are derived from, and clarify the separation of components from one another as development progresses.

In addition we describe three small 'pores' in the centre of the stomodeal anlage, similar to those observed in *L. geometricus *[37]. It is not currently understood what these pores are, but it has been proposed that their tri-radial construction may indicate a sister group relationship between pycnogonids (sea spiders) and chelicerates [40] and may therefore be of considerable phylogenetic value. The tools available for the study of *C. salei *will allow valuable future studies to increase understanding of this tri-radial structure.

We also include more detailed information about the later developmental processes of the embryo. For example, in parallel with the early differentiation of the brain parts, the germ band elongates by addition of more segments, and the whole embryo goes through a process of complex tissue movements known as inversion [30,41]. This process results in the enclosure of the dorsal yolk mass into the body of the embryo, and is accomplished by the dorsal migration of the right and left halves of the germ band that eventually meet in the dorsal midline (dorsal closure). To facilitate the precise mapping and description of developmental events during inversion, we divide the process into four stages (inversion I, II, III and dorsal closure).

Structures that undergo dramatic changes during inversion include the book lungs on the second opisthosomal segment and the tracheal system on the third opisthosomal segment, both of which are breathing organs. We observed fundamental differences in the sequence of appearance of the openings that give rise to the book lungs and the trachea, which we use to argue against the theory of serial homology of these organs [42].

## Results

### Embryonic stages

**Figure 1:**
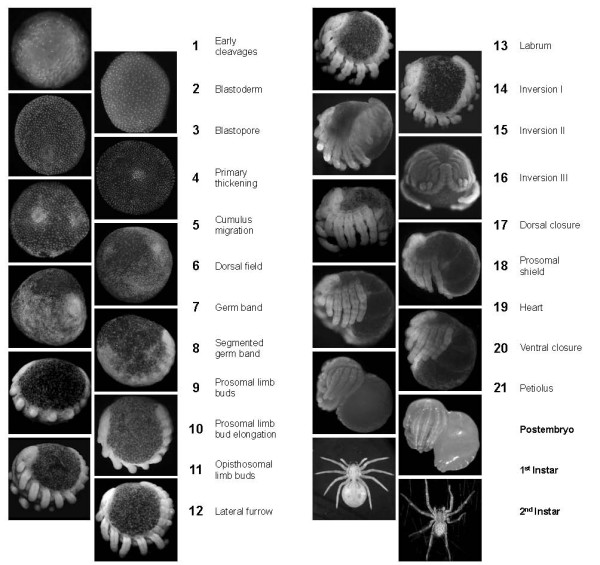
**Overview of development of the embryo, postembryo and first and second instar of *C. salei***. Nuclear stained embryos illustrate the 21 embryonic stages. The postembryo and the first and second instar are illustrated by images of live animals. Additional file 1 provides a comparison between this revised staging system and the system used in earlier publications.

The numbered stages into which we divide development in *C. salei *are intended to replace the existing hAEL staging system by Seitz [26]. Besides a number, each stage is also given a colloquial name for practical use. Figure 1 gives a general overview of our new system, and additional file 1 provides a detailed comparison between the old and new systems. The number of stages allocated to early development (up to stage 6) is higher in the Seitz system, where the early stages are separated into numerous time (hour) intervals after egg laying (hAEL). The resolution we chose for the early stages reflects the degree of detail we were able to observe using our methods, and follows the staging nomenclature of *A. tepidariorum *[25] until stage 9 (prosomal limb buds). From stage 6 to 10, the staging resolution of the two systems is similar, and after stage 10 our new system is more detailed (additional file 1).

#### Stage 1, Early cleavages

**Figure 2:**
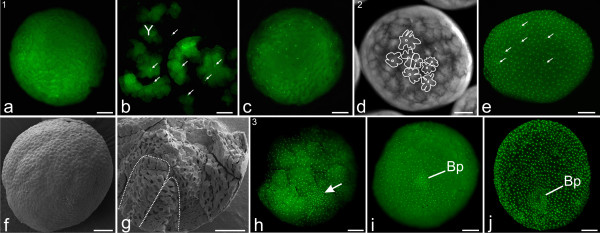
**Stages 1-3 of *C. salei***. All scale bars 200 μm. Sytox staining, **a-c**, **e**, **h-j**; light micrograph, **d**; SEMs, **f**, **g**. **a-c, Stage 1, Early cleavages**. **a**: The egg is spherical in shape, about 1.2 mm in diameter. Due to the large amount of yolk, the nuclei in the egg centre are not visible. **b**: Same egg as in **a**, broken apart. Nuclei (white arrows) are visible within the broken yolky mass (Y). **c**, More developed egg as in **a **with about 30 nuclei visible, still embedded in yolky mass. **d-e, Stage 2, Blastoderm**. **d**: In a live egg (egg number 2 from Additional file 2 at movie frame 76), the nuclei are visible as dark spots within the whitish periplasm. The periplasm is the primordial cytoplasm surrounding the nuclei and is lobate in shape (white lines) at the surface of the egg. **e**: Apart from some nuclei (white arrows) still surrounded by yolk only, most of the nuclei are enclosed by cell membranes, forming a cellular blastoderm. **f**: An egg (comparable to the egg in **e**) with a cellular blastoderm evident as a knobby surface texture. **g: **Parts of a broken egg at the same stage as the eggs in **e **and **f**. The yolky mass is now organized into large pyramid-shaped compartments (white dotted line). **h-j, Stage 3, blastopore**. **h**: A number of blastodermic cells aggregate and form the early blastopore (arrow). **i**: There are increasing numbers of cells in the blastopore (Bp) as it becomes more prominent. **j**: Slightly later, the blastopore (Bp) has a pore-like appearance.

During the process of egg-laying, the female spider produces a liquid secretion that guides the soft and ovoid-shaped eggs from the genital opening into a silk pouch that is then formed by the female into a round cocoon [7]. The liquid secretion is absorbed by the eggs, which as a result increase in size, become more solid and take on a spherical shape of roughly 1.2 mm in diameter. During these first hours of development the eggs are sticky and fragile. Because of these conditions, we waited 12 to 24 hours before it was possible to remove intact single eggs for further investigation. Eggs at stage 1 largely consist of yolk, which is distributed as fine homogeneous granules. In these early stages, the nuclei are surrounded by a mass of yolk and not yet enclosed within cell membranes. The first cycles of nuclear division are superficial (nuclear mitosis without cytokinesis which results in a polynuclear cell). The divisions take place intralecithally in the centre of the egg (Figures 2a, b). Based upon earlier observations [26] we assume that the first cleavage cycles are synchronous. During these cleavages the nuclei start to migrate towards the egg surface. This migration results in an egg with an even distribution of nuclei (Figure 2c).

#### Stage 2, Blastoderm

During stage 2, the cleavage energids (nuclei plus its surrounding cytoplasm) reach the egg surface where they are conspicuous with finger-like projections (Figure 2d, at movie frame 80 of Additional file 2). After a few division cycles, their cytoplasm attains a more rounded shape (at movie frame 210 of Additional file 2). This is when the cleavage type changes from superficial to holoblastic, and each nucleus on the egg surface is surrounded by its cell membrane (Figures 2e, f). A layer of early blastodermic cells is now evenly distributed over the egg surface (Figures 2e, f). The yolk mass is composed of compartments shaped like pyramids with the tips pointing towards the egg centre (white dotted line; Figure 2g). Data from nuclear staining and live-embryo imaging identify some nuclei that stay below the blastodermic cell layer (white arrows; Figure 2e). These nuclei probably belong to vitellophages; multi-nucleic cells which phagocytize the intracellular yolk. It is currently unclear whether all of these vitellophages have their origin in the early blastoderm (secondary immigration) or whether some of them derive from inside the egg [30,43]. However, it is likely that during cell migration from the egg centre to the surface, some cells remain in the yolk mass and do not reach the surface.

#### Stage 3, Blastopore

The following cell divisions are asynchronous. Because of the frequency of cell division cycles, the egg appears to contract (e.g. at movie frame 320 of Additional file 2). After roughly three cell division cycles, the contractions subside, leaving the blastodermic cells still more or less evenly distributed over the egg surface. There is no formation of a germ disc (a dense aggregation of cells that provides the primordial tissue for the embryo body and is commonly present in arthropod embryos [30]). However, in a particular region of the blastoderm cells begin aggregating to form the blastopore (white arrow; Figure 2h). Cells appear to migrate inwards at the blastopore (Figures 2h, i; at movie frame 200 of Additional file 3) and initiate gastrulation- the developmental process that results in a layer of mesendoderm beneath the surface layer of blastoderm (now ectoderm). As development progresses, the blastopore comprises more and more cells and becomes pore-like in appearance, while the surrounding blastoderm cells are no longer evenly distributed (Figure 2j).

#### Stage 4, Primary thickening

**Figure 3:**
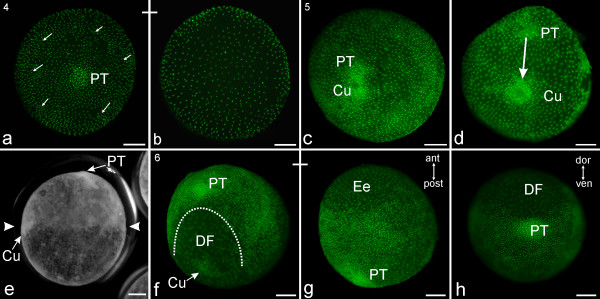
**Stages 4-6 of *C. salei***. All scale bars 200 μm. Sytox staining, **a-d**, **f-g**; light micrograph, **e**. **a-b, Stage 4**, **Primary thickening. a**: The blastopore region (evident in Figures 2**h-j**) consists of several dense layers of nuclei. It bulges slightly outwards, a phenomenon which in this and subsequent figures is called primary thickening (PT). The surrounding nuclei are arranged in irregular patches (arrows). **b**: Same egg as in **a**, seen from the opposite side. At this pole of the egg, the nuclei are more evenly distributed. **c-e, Stage 5, Cumulus migrating. c**: From the primary thickening, a large cell cluster (cumulus, Cu) starts to separate from the remaining rest of the primary thickening (PT). **d**: Due to the ongoing migration (white arrow) of the cumulus (Cu), the distance to the primary thickening (PT) increases. **e**: Living egg (egg number 1 from Additional file 2 at movie frame 960) in late stage 5. The cumulus (Cu) has reached its final position after separation from the primary thickening (PT). The migration of cells from the primary thickening beneath the surface layer has resulted in one hemisphere with greater cell density (top of photo **e**) compared with the other hemisphere (bottom of photo **e**). The line of demarcation between the dense hemisphere and the less dense one is the 'equator'. This marked difference in cell density is evident between the two arrowheads in **e**. **f-h, Stage 6, Dorsal field. f**: After the cumulus has reached the equator, it disintegrates. The tissue between the cumulus and the primary thickening spreads laterally, forming a region of low cell density called the dorsal field (DF). This region will continue to have much yolk in further stages of development. **g**: Same egg as in **f **rotated 120 degrees. The primary thickening (PT) is at the posterior edge of the developing embryo body and located in the hemisphere with greater cell density. The hemisphere with lower cell density is extra-embryonic (Ee) since it continues as a yolk-filled region. **h**: Posterior view of the primary thickening (PT). The dorsal field (DF) expands to about 100 degrees in width, and the cumulus has disappeared.

In the egg hemisphere that contains the advanced blastopore, scattered divisions result in an uneven distribution of the blastodermic cells. As a result, this half of the egg becomes more patchy (white arrows; Figure 3a). The blastodermic cells in the opposite egg hemisphere are evenly distributed though they appear to be fewer in number (Figure 3b). The blastopore region displays high nuclear density with the nuclei arranged in several layers. The pore-like composition of the blastopore disappears (Figure 3a). This indicates the end of gastrulation, i.e., the inward migration of cells at the blastopore. As development proceeds, the cellular tissue of the region where the blastopore formed becomes thicker and appears to bulge outwards (visible around movie frame 700 of Additional file 2). This conspicuous structure is known as the primary thickening, or cumulus anterior [25,31,32].

#### Stage 5, Cumulus migration

From the primary thickening (formerly the blastopore) clusters of internalised cells migrate in radial directions. Two different types of cellular movement are observed: The most prominent movement follows the asymmetrical fission of the primary thickening into two cell groups (visible in Additional file 2 at about movie frame 750 and in Additional file 3 at movie frame 570). A smaller group remains at the centre of the embryonic portion of the egg and continues to be identified as primary thickening. The larger cell group, the cumulus or cumulus posterior, is visible as a little bulge on the egg surface (Figures 3c-e; Additional files 2 and 3). The cumulus migrates about 90 degrees underneath the egg surface. The second type of cellular movement is a radial migration of single cells or groups of a few cells starting from the primary thickening. These cells (primordial mesendoderm) migrate directly underneath the ectodermal layer up to 90 degrees along the outer egg curvature (Additional files 2 and 3). At the end of the process, the migrating cells appear to be evenly distributed but are restricted to the egg hemisphere that has the primary thickening in its centre (Figure 3e).

**Figure 4:**
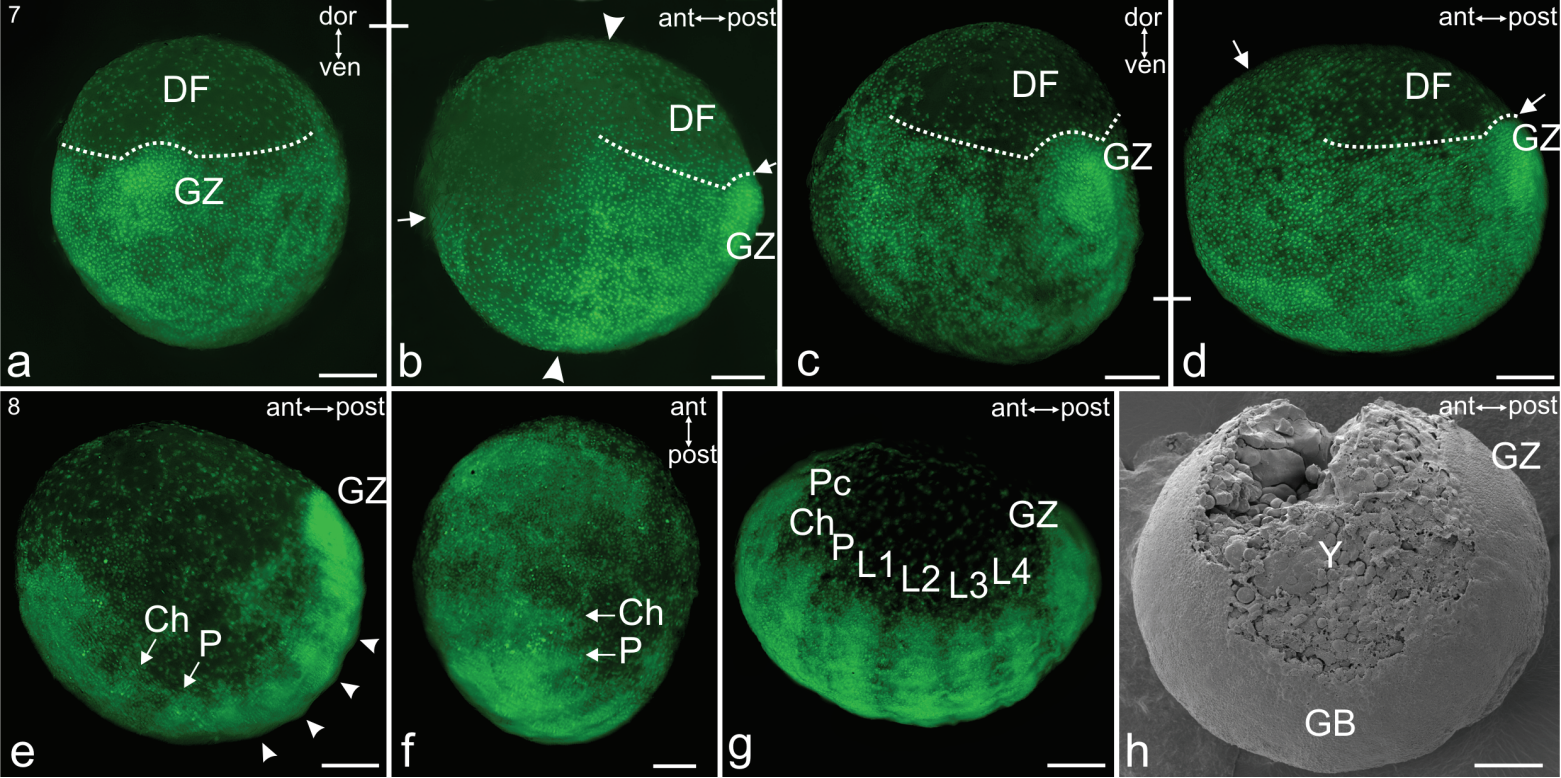
**Stages 7 and 8 of *C. salei***. Sytox staining, **a-g**; SEM, **h**. All scale bars 200 μm. **a-d, Stage 7, Germ band. a**: Posterior view of the growth zone (GZ), a dense region that is continuous with the primary thickening (PT) in stage 6, Figure 3**h**. The dorsal field (DF) is extended to its maximum. **b**: Same egg as in **a **rotated by 90 degrees, such that the embryo is in lateral view. The embryonic tissue (early germ band) extends along the ventral curvature between the two arrows. Between the two white arrow heads is the equator, which is the sharp change in cell density that marks the border of migration of mesendodermal cells from the primary thickening. **c**: Postero-lateral view of an embryo that is slightly more developed than the one in **a **and **b**. **d**: Lateral view of the same embryo as shown in **c**. The embryonic tissue (early germ band) extends along the ventral curvature between the two white arrows. This region has more cells and a much higher density than the dorsal field (DF). The equator is no longer visible. **e-h, Stage 8, Segmented germ band. e**: Lateral view. Evident are all future prosomal segments: cheliceres (Ch), pedipalps (P) and four walking legs (white arrow heads). At the posterior end, the growth zone (GZ) exhibits a higher density of cells. **f**: Frontal view of the same embryo as in **e**. **g**: Slightly more advanced embryo than **e**. Anterior to the cheliceral segment (Ch) the precheliceral region (Pc) is separated by a clear margin from the surrounding extra-embryonic (mainly yolk) tissue. All prosomal segments (cheliceres, Ch; pedipalps, P; four walking legs, L1-L4) are distinct. **h**: Lateral view. An embryo comparable to **e**. Evident are the germ band (GB) and yolk (Y). The yolk is located in the regions labelled earlier as the dorsal field (DF) and the extra-embryonic (Ee) region.

**Figure 5:**
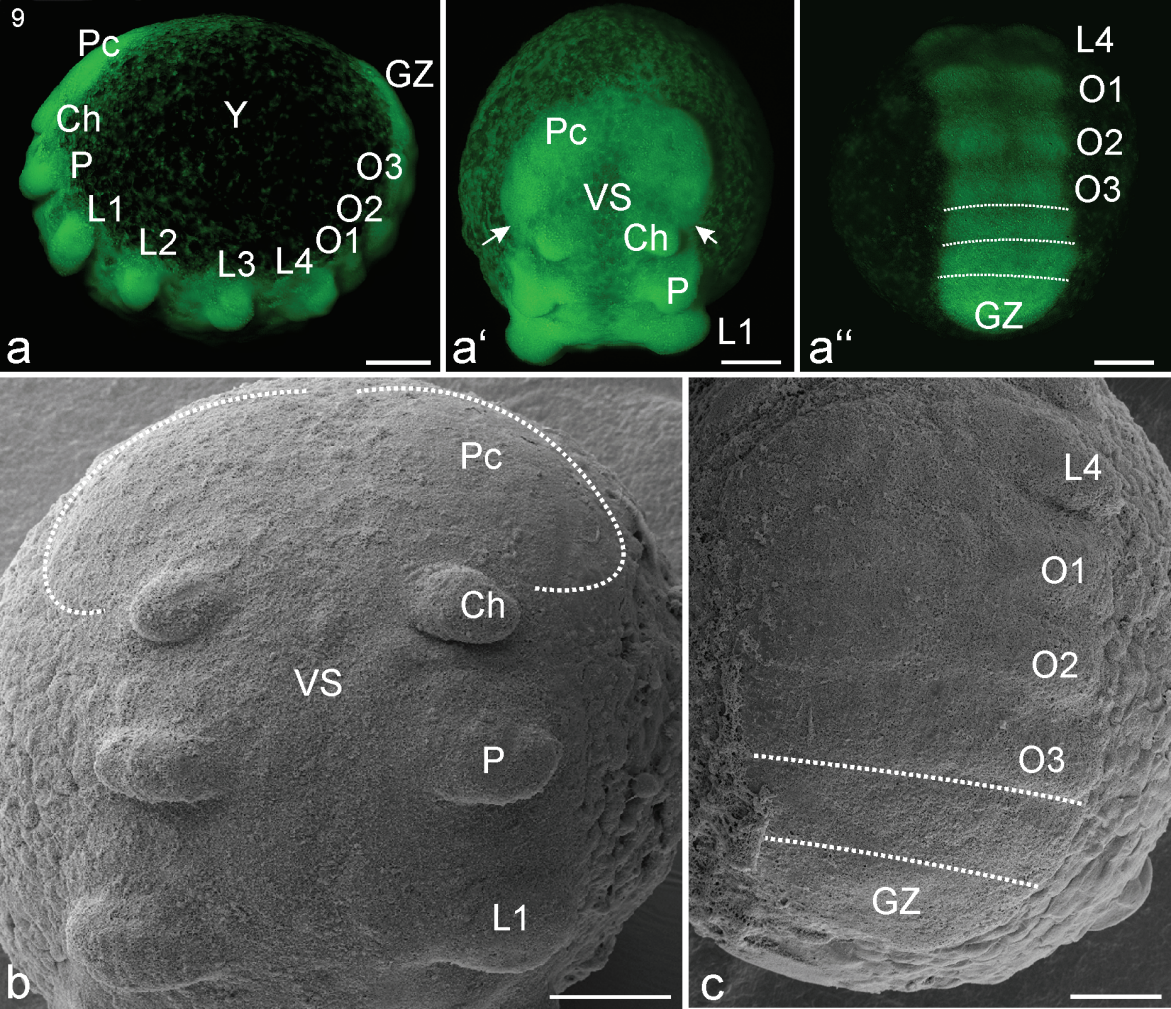
**Stage 9, Prosomal limb buds**. All scale bars 200 μm. Sytox staining, **a-a''**; SEMs, **b, c**. **a**: Lateral view of embryo. The dorsal field (DF) in earlier figures is probably now a mass of extra- and intra-cellular yolk (Y). Also evident are the precheliceral area (Pc), cheliceres (Ch), pedipalps (P), walking legs (L1-L4), opisthosomal segments (O1-O3) and the growth zone (GZ). **a'**: Frontal view. Between the developing appendages the ventral sulcus (VS) is visible as a narrow length of midline tissue with a low cell density compared with the bilateral appendage regions. The white arrows show that the precheliceral region (Pc) extends anteriorly from the anterior base of the cheliceres. **a''**: Posterior view. The dotted lines indicate the progress of opisthosomal segment formation anterior to the growth zone (GZ). **b**: Detail of the prosomal region in fronto-ventral view. The white dotted lines indicate the lateral margins of the precheliceral region (Pc). **c**: Detail of the opisthosomal region. Limb buds are barely evident at this stage on opisthosomal segments 1-3 (O1-O3). The white dotted lines indicate the progress of the formation of additional opisthosomal segments.

**Figure 6:**
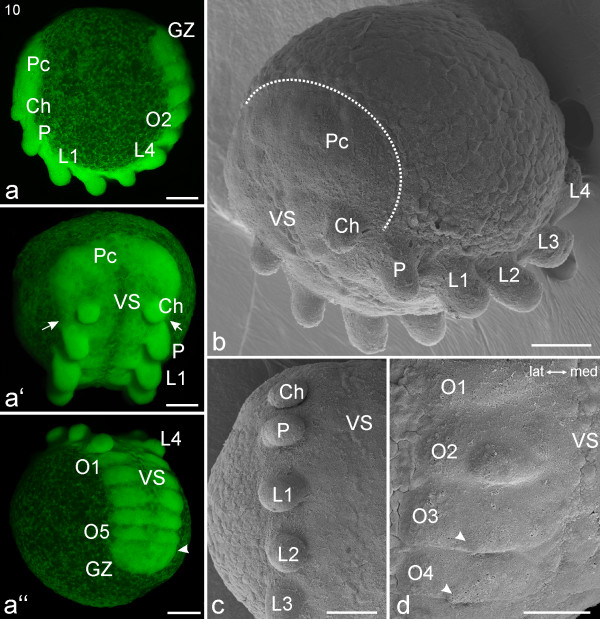
**Stage 10, Prosomal limb bud elongation**. All scale bars 200 μm. Sytox staining, **a-a''**; SEMs, **b-d**. **a**: Lateral view. Limb buds of pedipalps (P) and walking legs (L1-L4) are prominent in the prosoma, and segments are clearly distinguishable in the opisthosoma (e.g. segment two, O2). **a'**: Frontal view. The white arrows show that the precheliceral region (Pc) extends to the posterior base of the cheliceres (Ch). The ventral sulcus (VS) is a thin length of tissue between the germ band halves. **a''**: Posterior view. Five segments (O1-O5) with paired bulges are visible in the opisthosoma. A developing sixth opisthosomal segment (white arrowhead) is evident anterior to the growth zone (GZ). **b**: Embryo in fronto-lateral view. The white dotted line indicates the posterior border of the precheliceral region (Pc). **c**: Detail of the limb buds in the right prosomal region. **d**: Detail of the right opisthosomal region. The opisthosomal segment two (O2) has a clear limb bud while opisthosomal segments three and four (O3, O4) only show the beginning formation of the limb bud (white arrowheads).

The embryo now has two dissimilar egg hemispheres. The embryonic hemisphere appears to be more opaque because of the new layer of cells (primordial mesendoderm) underneath the ectoderm. The more translucent hemisphere is made up of extra-embryonic tissue, mainly filled with yolk in this and subsequent figures (e.g., Figures 3, 4, 5 and 6). The transition between the subsurface cell layer and the more translucent hemisphere has been called the 'equator' [26], and is clearly visible in living eggs (Figure 3e; Additional file 2). The cumulus is embedded at the edge of the equator and marks the anterior and dorsal region of the embryo body, while the primary thickening (in the centre of the embryonic hemisphere) is at the ventral and caudal pole of the embryo body (Figure 3e).

#### Stage 6, Dorsal field

This stage is characterised by a major rearrangement of embryonic tissue. Cells between the primary thickening and the cumulus start to migrate laterally, and the dorsal field is formed (DF, Figure 3f). This region is relatively translucent and is made up of far fewer cells than the ventral area where the embryo body will differentiate. Our data does not show whether the dorsal field is formed exclusively by cell migration or whether cell death is also involved. At the same time, the cumulus decreases in size (Figure 3f). As development progresses, the dorsal field expands approximately 100 degrees around the egg surface to form a semicircle, while the cumulus disappears (Figure 3h).

Figures 3g and 3h show that the embryo axes (anterior/posterior and dorsal/ventral) have now been specified as a result of preceding events (compare to [25]). The primary thickening (former blastopore region) will become the posterior end of the embryo body. Figure 3g is a ventral view showing the region of low cell density (extra-embryonic region) at the top of the photo, while the caudal primary thickening is at the bottom. Figure 3h is a posterior view of the dorsal field (DF) that becomes extra-embryonic.

#### Stage 7, Germ band

At this stage, it is not possible to determine the axis orientation of unstained eggs because of a lack of visible landmarks. When nuclear staining is applied, however, a germ band becomes evident (Figures 4a-d). The germ band is a ventral strip of cells with a convex flexion, bearing the former primary thickening at its posterior end (Figure 4h). This latter region is now called the growth zone (GZ) as in Figures 4a-d. The embryonic tissue expands anteriorly beyond the ventral equator margin (between the white arrows; Figure 4b).

As the germ band becomes visible and gradually lengthens along the ventral curvature, the equator disappears and the dorsal field expands laterally. The equator is evident in Figure 3e (between the white arrow heads) and Figure 4b (between the white arrow heads) but is not evident in Figures 4c, d and later figures. The widening of the dorsal field is evident (white dotted lines) in Figures 4b-d. At the end of this 'Germ band' stage, the embryonic tissue has lengthened anteriorly and covers the entire ventral surface of the embryonic region. In this ventral region of the embryo, the cell density is much greater than in the dorsal field. As a result of cell migration, the cells in the embryonic region become more evenly distributed with only the dorsal field displaying a significantly lower cell density (DF; Figure 4d).

#### Stage 8, Segmented germ band

Within the spherical eggshell, the embryo now has the shape of a flattened ovoid. The C-shaped germ band invariably lies along the longitudinal egg axis (Figures 4e-h). The developing embryo body (embryo proper) of primordial segments and appendages is very dense, comprising many more cells than the dorsal extra-embryonic tissue (Figures 4e-g). The dense region of cells just anterior to the cheliceres initially has no organized structures (Figures 4e, f) and the border between this region and the extra-embryonic region is less defined than the perimeter of the rest of the embryo body. This precheliceral region (Pc) gradually becomes consolidated into a more distinct structure (Figures 4g, 5a, a'). All future prosomal segments (those of the cheliceres, pedipalps and four walking legs) are visible and distinctly divided by inter-segmental furrows (Figure 4g). The cheliceral segment is slightly smaller than the posterior segments (Figures 4e-g). At its posterior end, the embryo proper has a growth zone (GZ; Figures 4e, g, h) which appears brighter in nuclear staining. The growth zone is rounded posteriorly and has a higher cell density than the remaining embryo proper.

#### Stage 9, Prosomal limb buds

At this stage (Figure 5a) the border of the embryo proper is more clearly defined than during the previous stage. In the precheliceral region there is often a slight difference in the progression of development of the right and left halves (e.g. Figure 5a'). The lower cell density in the medial precheliceral region marks the start of the formation of the ventral sulcus (VS; Figure 5a') which continues to extend posteriorly into the midline of the anterior segments (cheliceres and pedipalps) of the prosoma (Figures 5b, 6a'). The posterior margins of the precheliceral lobes appear anterior to the cheliceral segment but will gradually extend posteriorly (white arrows; Figures 5a', b). All the prosomal segments are more prominent than in the previous stage, and the prosomal limb buds (cheliceres, pedipalps and four walking legs) bulge outward. The buds are broad and flat and point in a postero-ventral direction (Figures 5a, a', b). The cheliceral buds are smaller and slightly more medial than the buds of the pedipalps and walking legs (Figures 5a, a', b). The opisthosoma has about four visible segment anlagen (Figures 5a, a'', c). The growth zone has a posterior curvature and is less broad than the more anterior segments (Figures 5a'', c).

#### Stage 10, Prosomal limb bud elongation

For stages 1-9 we adhere to the staging system defined for *A. tepidariorum *[5,25,44]. However, we deviate from this system at stage 10 as this stage is not well described for *A. tepidariorum *and has not been used extensively by other authors. Furthermore, stage 10 for *A. tepidariorum *represents too great an advance from stages 1-9. We define a new stage 10 and new subsequent stages for *C. salei*. In our stage 10, the embryo has reverted from an ovoid to a largely spherical shape (Figure 6a). The precheliceral region is broader than the remaining parts of the germ band (Figures 6a', b). The posterior margins of the precheliceral region have moved anterior to the pedipalpal segment and now enclose the cheliceral segment (Figures 6a', b).

None of the prosomal limb buds are segmented yet, and they vary in their width-to-length ratio (Figures 6a-c). The slightly depressed ventral sulcus (VS) extends from the centre of the precheliceral region to opisthosomal segment three (Figures 6a', c). The first opisthosomal segment is clearly visible but will eventually disappear (Figure 6d). It is at this point considerably smaller than the subsequent opisthosomal segments. In Figure 6a'' a developing sixth opisthosomal segment (white arrow head) is evident anterior to the growth zone. In older embryos of this stage, limb buds appear on opisthosomal segment two (Figure 6d).

#### Stage 11, Opisthosomal limb buds

**Figure 7:**
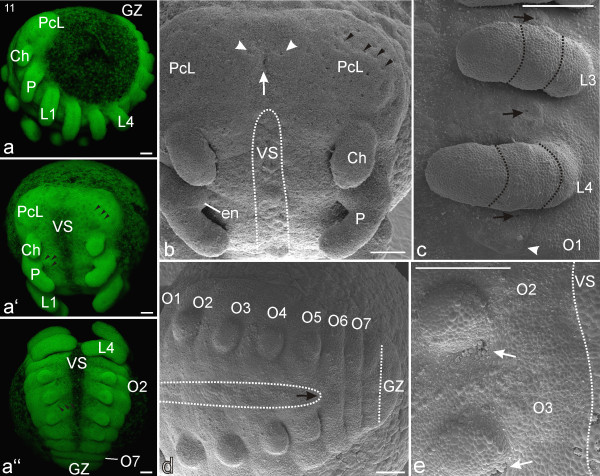
**Stage 11, Opisthosomal limb buds**. All scale bars 100 μm. Sytox staining, **a-a''**; SEMs, **b-e**. **a**: Lateral view. The single precheliceral area (Pc, Figure 6) is now divided into bilateral precheliceral lobes (PcL). **a'**: Frontal view. Laterally adjacent to the ventral sulcus, segmentally iterated point-like depressions are evident (black arrow heads). This is presumably neural precursor tissue. **a''**: Posterior view. The ventral sulcus (VS) is prominent between the developing limb buds in the prosoma and opisthosoma. **b**: Frontal view clearly showing that the single precheliceral area in earlier figures is now divided into bilateral precheliceral lobes (PcL). Each of these lobes has evenly distributed point-like depressions (some indicated by black arrow heads). The white arrow shows the postero-medial furrow of the forming stomodeum. Laterally adjacent to it are two conspicuous point-like neural depressions (white arrow heads). At the pedipalps (P), the anlage of an endite (en) is evident as a proximo-medial swelling. **c**: Detail of left prosomal region. Prosomal limb buds (L3-4) appear three-segmented, and in between the limb buds is a larger invaginating region (black arrows) of presumptive neural precursor cells. Barely visible are the remnants of limb buds (white arrow head) on opisthosomal segment one (O1). **d**: Opisthosomal region. Seven clearly separated opisthosomal segments (O1-O7) are visible while an additional segment (vertical dotted line) is still connected with the growth zone (GZ). The ventral sulcus (VS, horizontal white dotted lines) extends posteriorly (black arrow) as the limb buds become differentiated **e**: At the medio-posterior base of the limb buds of opisthosomal segments two and three (O2, O3) are conspicuous depressions (white arrows) made up of primordial tissue for the respiratory system. The white dotted line indicates the right boundary of the ventral sulcus (VS). Ch: chelicere, P: pedipalp.

At this stage, the precheliceral region is partitioned medially into bilateral precheliceral lobes (Figures 7a', b). Each lobe has about 30 point-like depressions (invagination sites, *sensu *[14]) that are presumably neural precursor tissue (black arrow heads, Figure 7b). The anlage of the stomodeum becomes visible. It is formed by a somewhat depressed antero-medial precheliceral region, which bears a small longitudinal furrow with two lateral adjacent invaginating neural precursor groups (Figure 7b).

In older embryos of this stage, the stomodeal anlage has migrated posteriorly, leaving behind a shallow cleft between the precheliceral lobes (Figure 7b). The cheliceral limb buds have become more flattened and are approximately twice as long as they are wide. They have twisted slightly ventro-posteriorly, and their distal parts are cone-like (Figures 7a', b). The pedipalps have a proximo-medial swelling formed by the anlage of an endite (en, Figure 7b). The ventral sulcus has extended posteriorly, reaching the sixth opisthosomal segment in older embryos of this stage (Figures 7a'', d). Small spots (black arrow heads; Figures 7a', a'') in a segmentally iterated pattern are barely visible laterally adjacent to the ventral sulcus. This area is the ventral neuroectoderm and the point-like depressions correspond to neural precursor tissue [27]. In addition, medially and between the developing limb buds, larger spots of neural precursor tissue are observable on the ventral surface of each prosomal segment (black arrows, Figure 7c). Together with the point-like depressions of the precheliceral region (black arrow heads; Figure 7b) they are the first external indications of neurogenesis.

The pedipalps and walking legs continue elongation and start to bend ventrally. Two annulations develop, dividing the limb buds into three regions (black dotted lines; Figure 7c). For a short time, a small structure is visible on the first opisthosomal segment. This small structure can be interpreted as a vestige or remnant of an appendage (white arrow; Figure 7c). With a developmental gradient from anterior (more developed) to posterior (less developed), primordial limb buds have appeared as small bulges on opisthosomal segments two to five (Figures 7a, a'', d). The initial shape of these buds is not as broad as the prosomal limb buds when they first appeared (compare with stage 9; Figures 5a', b). Depressions at the medio-posterior insertion of opisthosomal limb buds two and three can be seen (white arrows; Figure 7e). These invaginations are precursor tissue for the book lung system. At least seven separated segments are visible anterior to the growth zone (Figures 7a'', d).

#### Stage 12, Lateral furrow

**Figure 8:**
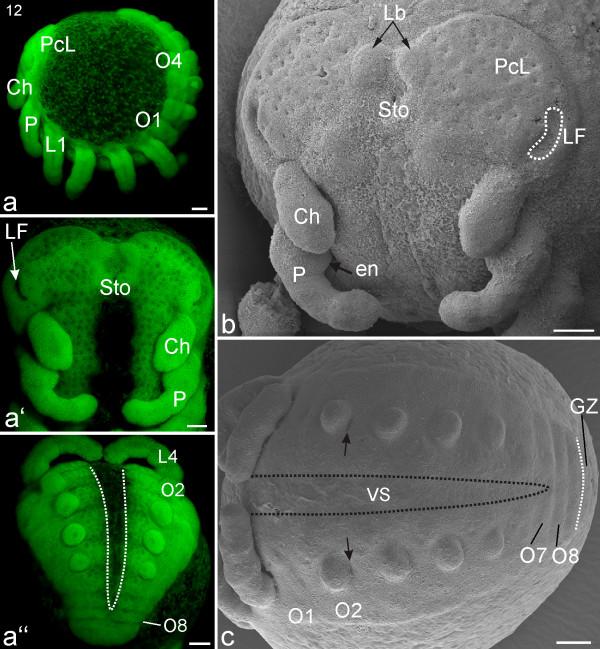
**Stage 12, Lateral furrow**. All scale bars 100 μm. Sytox staining, **a-a''; **SEMs, **b**, **c**. **a**: Lateral view. **a'**: Frontal view. Lateral to the stomodeum (Sto) are invaginating folds of precursor neural tissue (LF, *lateral furrow*). The pedipalps (P), cheliceres (Ch), and stomodeum are more pronounced. **a''**: Posterior view. The walking legs (visible L4) are more elongated and their tips touch each other. The ventral sulcus (VS, white dotted line) extends posteriorly almost the eighth opisthosomal segment (O8). **b**: Ventro-lateral view. Anterior to the stomodeum (Sto), the small bi-lobed anlage of the labrum (Lb) is visible. Postero-laterally on each precheliceral lobe (PcL), a kidney shaped *lateral furrow *(LF) is evident. **c**: Opisthosomal region. Eight separate opisthosomal segments (O1-O8) are visible whilst an additional segment (indicated by a white dotted line) is still connected to the growth zone (GZ). Small depressions (black arrows) of the primordial respiratory system are evident at the posterior insertion of the limb bud at opisthosomal segment two (O2). The ventral sulcus (VS, black dotted line) extends posteriorly to the seventh opisthosomal segment (O7).

The outer edges of the precheliceral lobes have become very distinct, and the lobes stand out clearly from the surrounding tissue (Figures 8a', b). Alongside the point-like depressions, a slight relief begins to form on the precheliceral lobes. Lateral to the stomodeum, invaginating kidney shaped folds of neural tissue are visible (*lateral furrow *LF; Figures 8a', b). Medially adjacent to these folds a minimal elevation can be seen in some specimens.

The stomodeum has now subsided and moved further posteriorly so that the cleft separating the precheliceral lobes is more evident (Figure 8b). Anterior to the stomodeum, the small bi-lobed anlage of the labrum is visible (Lb; Figure 8b). The prosomal limbs (pedipalp and walking legs) have elongated, and the tips of the walking legs from each body halve approach each other. The pedipalpal endite on the most proximal segment (coxa) is clearly visible (en; Figure 8b). The pedipalps and all the walking limbs display signs of annulations. It is not clear how these annulations relate to later leg segments.

The ventral sulcus extends posteriorly to the seventh opisthosomal segment, and has slightly widened (Figures 8a'', c). All opisthosomal limb buds have a globular shape (Figures 8a'', c). Small depressions of the primordial respiratory system are evident at the posterior insertion of the limb bud on opisthosomal segment two (black arrows; Figure 8c). Medially and between the opisthosomal limb buds, large point-like depressions of neural precursor tissue can be seen. These depressions are similar to the large spots on the prosoma in stage 12 (compare Figure 8c with Figure 7c). Up to eight separate opisthosomal segments are visible anterior to the growth zone (Figures 8a'', c).

#### Stage 13, Labrum

**Figure 9:**
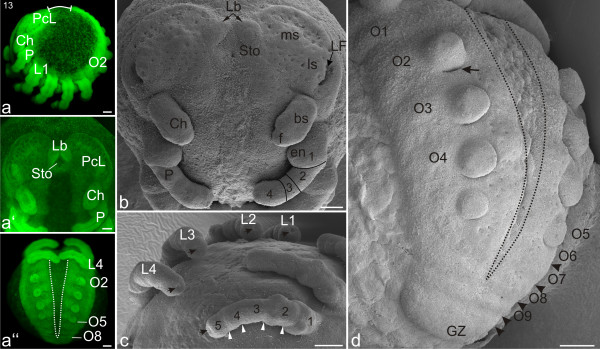
**Stage 13, Labrum**. All scale bars 100 μm. Sytox staining, **a-a''**; SEMs; **b-d**. **a**: Lateral view. Cheliceres (Ch), pedipalps (P) and the four walking limbs (L1-L4) are more prominent in the prosoma. Because of the growth that has taken place along the ventral curvature, the posterior end of the opisthosoma approaches (white line) the anterior border of the precheliceral lobes (PcL). **a'**: Frontal view. Anterior to the stomodeum (Sto) the prominent labrum (Lb) is evident. Cheliceres (Ch) and pedipalps (P) are more pronounced. **a''**: Posterior view. The ventral sulcus (VS, white dotted line) extends posteriorly to the eighth opisthosomal segment (O8). **b**: Precheliceral region. Two distinct fields of precursor neural tissue are evident within the crescent-shaped precheliceral lobes: the *medial *(ms) and *lateral *(ls) *subdivisions*. The cheliceres (Ch) have a proximal base (bs) and a distal fang (f). The pedipalp (P) is four-segmented (1-4) and bears an endite (en) on its first segment. **c**: Prosoma in postero-lateral view. The white arrowheads show that each walking leg (L1-L4) is subdivided into five podomeres (1-5). There is a point-like depression (black arrows) of what is presumed to be neural precursor tissue at the distal tip of each leg. **d**: Opisthosomal region. Nine opisthosomal segments (O1-O9) are visible while an additional segment is still connected to the growth zone (GZ). Black arrowheads indicate segmental furrows that mark the boundary between opisthosomal segments. A slit-like invagination (black arrow, primordial respiratory tissue) is evident at the posterior base of the limb bud at opisthosomal segment two (O2). The ventral sulcus (black dotted line) extends posteriorly to the eighth opisthosomal segment (O8).

The distance between the posterior end of the opisthosoma and the anterior border of the precheliceral lobes is at its smallest at this stage (white line; Figure 9a). In the forming brain, the *lateral furrows *have deepened (Figure 9b). Two distinct fields of neural precursor tissue are evident within the crescent-shaped precheliceral lobes: the *medial subdivision *and the *lateral subdivision *(*ms *and *ls, sensu *[37]). These are positioned between the *lateral furrow *and the anlage of the labrum (Figure 9b). The two lobes of the labrum are clearly evident at this stage (Figure 9b) but are still separate structures (compare with later stages; e.g. Figure 12 b).

The cheliceres have a proximal base and the anlagen of the distal fangs are visible (f; Figure 9b). The pedipalps and walking legs show clearer annulations and a subdivision into podomeres is evident. The pedipalps are divided into four segments, and the walking legs have five segments (Arabic numbers in Figures 9b, c). The most proximal leg segments (coxa and trochanter/femur) are broader than the more distal leg segments (Figure 9c). Large invagination sites are positioned at the distal tips of the pedipalps and walking legs (black arrows; Figure 9c).

All opisthosomal limb buds retain their globular shape. A slit-like invagination is evident at the posterior base of the limb bud on opisthosomal segment two (black arrow, Figure 9d) and will be the first opening of the primordial respiratory tissue (book lung system). The fifth opisthosomal limb buds are still smaller than the more anterior buds. The opisthosoma has up to nine separated segments and the ventral sulcus, which has again slightly widened, extends posteriorly to the eighth opisthosomal segment (Figures 9a, d).

#### Stage 14, Inversion I

**Figure 10:**
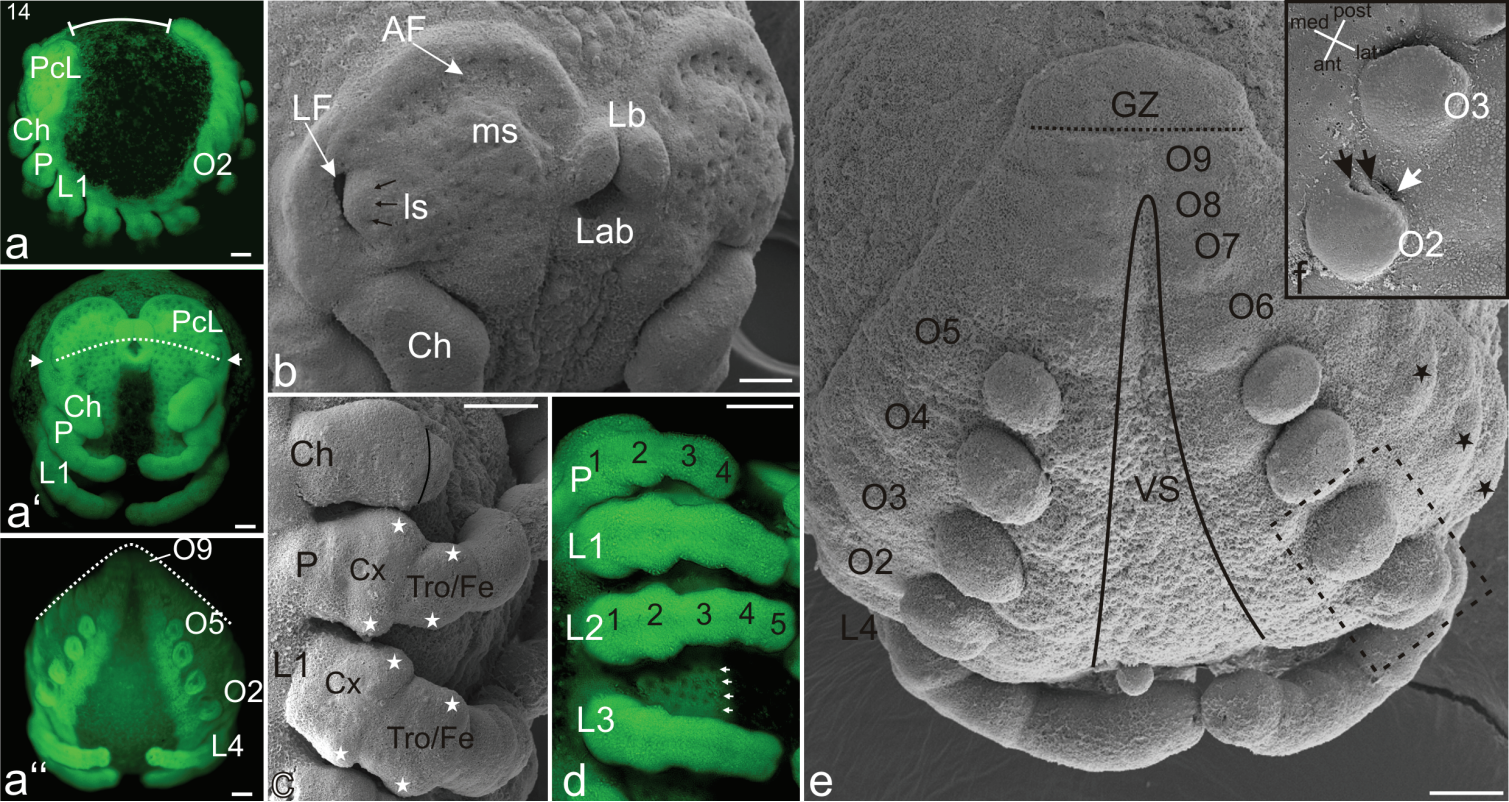
**Stage 14, Inversion I**. All scale bars 100 μm. Sytox staining, **a-a''**, **d**; SEMs, **b**, **c**, **e**, **f**. **a**: Lateral view. The distance between the posterior opisthosoma and the anterior border of the precheliceral lobes (PcL) has increased (indicated by white line, compare with Figure 9**a**). **a'**: Frontal view. Between the precheliceral lobes (PcL) the stomodeum (Sto) has moved posteriorly. The white dotted line shows the more anterior position of the mouth opening in relation to the *lateral subdivision *(indicated by white arrows) of the brain. **a''**: Posterior view. The white dotted line shows the progress of inversion (see the upper diagram in Figure 11**d **which schematically illustrates inversion). **b**: Head in ventro-lateral view. Anterior to the *medial subdivision *(ms), the *anterior furrow *(AF) has formed. The *anterior furrow *has also been termed semi-lunar or cerebral groove in other arachnids [e.g. [33,45]]. The *lateral subdivision *(ls) migrates (black arrows) in the direction of the *lateral furrow *(LF), partly covering it. The mouth opening is surrounded anteriorly by the labrum (Lb) and posteriorly by the labium (Lab). **c**: Lateral view of right prosomal region. The most proximal limb segments (coxa, Cx and trochanter/femur, Tro/Fe) of the pedipalp and each prosomal limb are widened in anterior-posterior direction (white stars). **d**: Lateral view of right prosomal region. The ectodermal tissue medial to the prosomal limbs shows a grid-like formation of black spots (white arrows), presumably primordial neural tissue. **e**: Opisthosomal region. The black line indicates the relative progress of the ventral sulcus (VS). Nine separate opisthosomal segments are present. The black dotted line indicates an additional segment anterior to the growth zone (GZ). The black asterisks designate lobes of anlagen that will eventually become tergites on the dorsal surface of the body. **f**: Detail of right limb buds of opisthosomal segments two and three (for orientation see dashed-line box in **e**). At the lateral base of the limb buds of opisthosomal segment two (O2), the opening of the pulmonary sac (white arrow) can be seen. Medially adjacent to it are two slit-like openings (black arrows) to the developing book lungs. Ch, chelicere; L1-L4, walking legs one to four; P, pedipalp.

**Figure 11:**
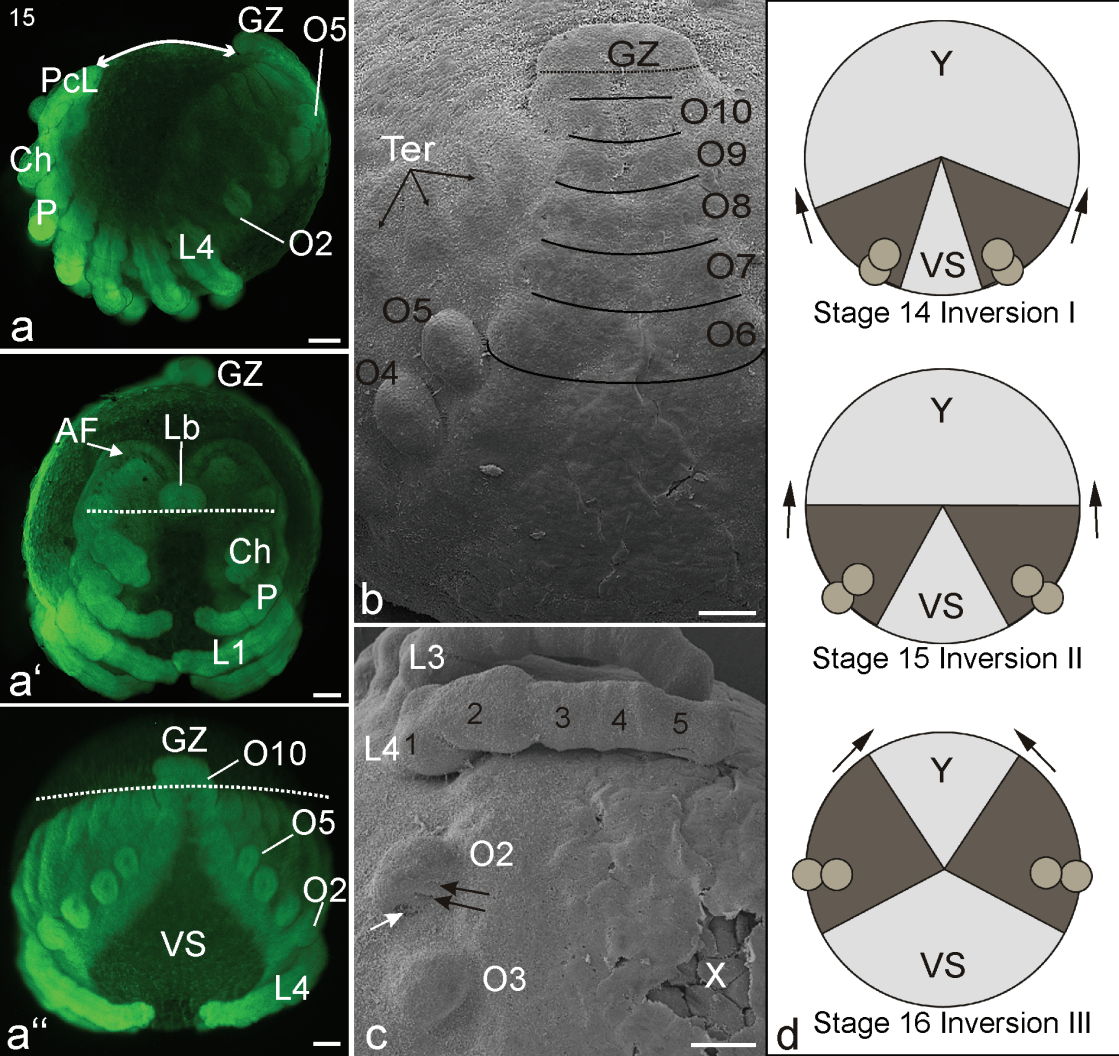
**Stage 15, Inversion II**. All scale bars 100 μm. Sytox staining, **a-a''**; SEMs, **b**, **c**. **a**: Lateral view. The white line indicates the increased distance from the precheliceral lobes (PcL) to the opisthosomal tail compared with previous stages (see Figure 10**a**). **a'**: Frontal view. The white dotted line indicates the mouth opening between the two lateral subdivisions of the developing brain (compare with Figure 10**a'**). The two labral lobes have completely fused and the labrum (Lb) is now an unpaired structure. **a''**: Posterior view. The white dotted line shows the progress of inversion (middle diagram in **d**). **b**: Opisthosomal region. Separated opisthosomal segments four to nine (O4-9) are visible. The tenth (O10) and the future eleventh segments (black dotted line) are located together with the growth zone (GZ) in a tail-like portion of the germ band that protrudes from the mass of yolk. Small bulges of tergite anlagen (Ter) are evident on the dorsal surface (compare with the more differentiated tergite anlagen in Figure 14**c**). **c**: Detail of the right third and fourth walking legs (L3, L4) and the limb buds of opisthosomal segments two and three (O2, O3). At the posterior base of the limb bud of O2 the opening of the pulmonary sac (white arrow) is seen, and adjacent to it medially are two slit-like openings (black arrows) to the book lungs. The podomeres of the fourth walking leg (L4) are numbered (1-5) from base to tip. **d**: Schematic illustration of the steps of inversion corresponding to stages 14 (compare with Figure 10 **a''**), 15 (compare with Figure 11 **a''**), and 16 (compare with Figure 12 **a''**). Posterior view, dorsal is at the top of the diagrams. The germ band (brown areas) has divided, and the ventral sulcus (VS) is increasing in width. The bilateral regions of the germ band are migrating dorsally (black arrows), enclosing the yolk area (Y) and eventually meeting in the dorsal midline (stage 17, dorsal closure). By stage 15, the dorsal edges of both halves of the germ band lie in a line when viewed from posterior. AF, anterior furrow; Ch, chelicere; P, pedipalp; X, damaged area, cuticle torn.

The gradual widening of the ventral sulcus, which from stage 11 to 13 is a relatively slow process, significantly accelerates during stage 14 (Figures 10a'', e). This marks the start of inversion, a complex sequence of tissue movement and growth that results in a rearrangement of the body and incorporation of the yolk mass into the embryo. Apart from the precheliceral region and the posterior-most opisthosomal segments, the two halves of the germ band move separately over the yolk mass until they connect again on the dorsal side (Figure 11d gives a schematic overview). As a result of this movement, the distance between the precheliceral region and the posterior opisthosomal region increases. Simultaneously, the germ band continues to extend with the addition of the final opisthosomal segments. The precheliceral region, which until inversion was an extension of the rest of the germ band, gradually folds posteriorly. In order to precisely map the various developmental events that occur during inversion, we distinguish four separate stages.

At Inversion I, the dorsal edges of the body halves have not yet reached the upper hemisphere of the egg. The precheliceral lobes are characterized by a high density of point-like depressions and even more pronounced anterior rims (Figures 10a', b). In addition, the *medial *and *lateral subdivisions *are more evident. Anterior to the *medial subdivision*, a crescent shaped *anterior furrow *has formed (AF, Figure 10b). The *anterior furrow *has also been termed the semi-lunar or cerebral groove in other arachnids [e.g. [33,45]]. The *lateral subdivision *migrates in the direction of the *lateral furrow*, partly covering it (black arrows; Figure 10b). The cheliceres are now two-segmented. The proximal segment (basal segment) widens distally and the tapering distal segment (fang) sits slightly off-centre on the basal segment (Figure 10c). The proximal segments (coxa and trochanter/femur) of the pedipalps and walking legs are wider than the more distal segments (white stars; Figure 10c). This widening is probably related to anterior and posterior invagination sites on each of these leg segments. The neuroectoderm medial to the prosomal limbs displays a grid-like formation of point-like depressions (white arrows; Figure 10d).

The buds on opisthosomal segment two have become dorso-ventrally elongated. On the posterior ends of these buds, the opening of the pulmonary sac and one or two pulmonary furrows are evident (Figures 10e, f). The buds on opisthosomal segments three to five are undifferentiated and still more or less globular in shape. Dorsal to the opisthosomal limb buds, the anlagen of the tergite plates are evident (black stars; Figure 10e). At the posterior end of the embryo, nine opisthosomal segments have separated from the growth zone (Figures 10a, e). The growth zone now protrudes slightly from the yolk, marking the start of the tail-like formation of the 'post-opisthosoma' (name derived from '*Postabdomen' *[46]).

#### Stage 15, Inversion II

By the second stage of inversion, the lateral/dorsal movement of the body halves has progressed, and the anlagen of tergite plates have extended dorsally (Figures 11a'', d). The opisthosomal body halves have reached the dorsal hemisphere of the egg and form a line when viewed from a caudal perspective (white dotted line; Figure 11a''). The two labral lobes have completely fused and the labrum is now an unpaired structure (Figure 11a'). The labrum and stomodeum have jointly started the posterior migration that will be continued in subsequent stages. By stage 15, the cleft between the two precheliceral lobes has become deeper, and the mouth opening lies between the *lateral subdivisions *on both head lobes (white dotted line; Figure 11a'). The labrum now partially covers the stomodeum (Figure 11b).

Ten separate opisthosomal segments are evident anterior to the growth zone (Figures 11a'', b). The posterior base of the limb bud on opisthosomal segment two bears two pulmonary furrows (black arrows) and the lateral opening of the pulmonary sac (white arrow; Figure 11c). The tenth and the future eleventh opisthosomal segments are now forming, together with the growth zone of the tail-like post-opisthosoma (Figure 11b). Small bulges of tergite anlagen are evident on the dorsal surface of the opisthosomal segments (Figure 11b).

#### Stage 16, Inversion III

**Figure 12:**
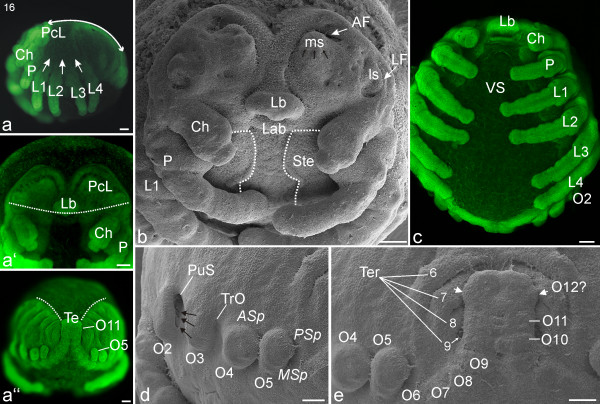
**Stage 16, Inversion III**. All scale bars 100 μm. Sytox staining, **a-a''**, **c**; SEMs, **b**, **d, e**. **a**: Lateral view. The white line indicates the increased distance from the precheliceral lobes (PcL) to the opisthosomal tail compared to previous stages (compare with Figure 10**a **and 11**a**). The prosomal tergites start to extend dorsally (white arrows). **a'**: Frontal view. The white dotted line indicates the more posterior position of the mouth opening in relation to the *lateral subdivision *of the brain (compare with Figures 10**a'**and 11**a'**). **a''**: Posterior view. The white dotted line shows the progress of inversion (see the lower diagram in Figure 11**d **which schematically illustrates inversion). **b**: Detail of the head region. The *medial subdivision *(ms) is growing anteriorly (black arrows), partially covering the *anterior furrow *(AF). The *lateral furrow *(LF) is totally covered by tissue from the *lateral subdivision *(ls). The mouth opening is covered by the medially enlarged tip of the labrum (Lb). Anlagen of the segmental sternites (Ste, white dotted line) are evident medial to the pedipalps (P) and walking legs (L1). **c**: Ventral view showing all prosomal appendages and the extent of the widening of the ventral sulcus (VS). **d**: Detail of left anterior opisthosoma. At the posterior base of the limb bud on opisthosomal segment two (O2), three pulmonary furrows (black arrows) and a lateral opening of the pulmonary sac (PuS) are evident. At the latero-posterior insertion of the limb bud on opisthosomal segment three (O3), the opening of the tubular trachea (TrO) is visible. The globular limb bud on opisthosomal segment four (O4) will differentiate into the anterior spinneret (*ASp*), whereas the dorso-ventrally elongated limb bud on opisthosomal segment five (O5) will differentiate into the posterior (*PSp*) and medial (*MSp*) spinnerets. **e**: Detail of the posterior opisthosomal region. Eight opisthosomal segments (O4-O11) are clearly evident here. On the dorsal surface, the primordial tergite plates (Ter) have further expanded (compare with the later stage in Figure 14**c**). Between the eleventh opisthosomal segment (O11) and the growth zone (GZ), small bilateral lobes probably represent the twelfth opisthosomal segment (O12?). Ch, chelicere; Lab, labium; Te, telson.

By the third stage of inversion, the tergite plates of the opisthosoma are completely enclosed within the dorsal hemisphere of the egg: from a caudal perspective the opisthosomal limb buds of both halves lie more or less in one line (Figures 12a'', 11d). The distance between the precheliceral lobes and post-opisthosoma has increased and is about a quarter of the total circumference of the embryo (Figure 12a). The *lateral furrows *are completely covered by tissue from the kidney-shaped *lateral subdivisions *(Figure 12b). The *medial subdivisions *are growing anteriorly, partially covering the *anterior furrows *(black arrows; Figure 12b). The *anterior furrows *are partially closed by anterior expansions of *medial subdivisions *(Figure 12b). The tip of the labrum is stretched medially and points in a ventral direction (Figures 12a', b). Posterior to the stomodeum, the unpaired anlage of the labium is formed (Figure 12b). The mouth area (labrum, stomodeum and labium) has migrated further and lies posterior to the *lateral furrow/lateral subdivision*, on a level with the insertion of the cheliceres (white dotted line; Figure 12a'). The bases of the cheliceres have further widened, and at the ventral base of the pedipalp a prominent endite is evident (Figure 12b). The coxae of the pedipalps and walking legs still have a 'bi-lobed' appearance, and these appendages have elongated (Figures 12a, c). Anlagen of the segmental sternites become visible medial to the pedipalps and walking legs (white dotted line: Figure 12b). The prosomal tergites start to extend dorsally (white arrows; Figure 12a).

The posterior base of the limb bud on opisthosomal segment two bears three pulmonary furrows (black arrows) and the lateral opening of the pulmonary sac (PuS, Figure 12d). At the latero-posterior insertion of the limb bud on opisthosomal segment three, the invagination of the tubular trachea is visible (white arrow; Figure 12d). The globular limb bud on opisthosomal segment four will eventually differentiate into the anterior spinneret (*ASp*), while the dorso-ventrally elongated limb bud on opisthosomal segment five will differentiate into the posterior (*PSp*) and medial (*MSp*) spinnerets (Figure 12d).

On the dorsal surface, the opisthosomal tergite plates have further expanded, and their dorsal edges start to approach each other (Figures 12a'', e). Eleven opisthosomal segments have formed anterior to the growth zone. In between the eleventh opisthosomal segment and the growth zone (GZ), small bilateral lobes probably represent the twelfth opisthosomal segment (arrowheads; Figure 12e).

#### Stage 17, Dorsal closure

**Figure 13:**
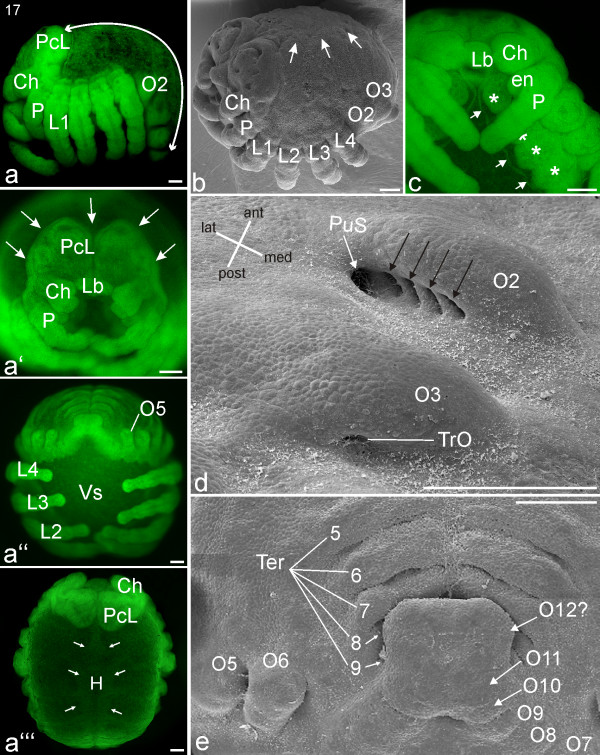
**Stage 17, Dorsal closure**. All scale bars 100 μm. Sytox staining, **a-a'''**, **c**; SEMs, **b**, **d, e**. **a**: Lateral view. The white line indicates the increased distance from the precheliceral lobes (PcL) to the opisthosomal tail compared to previous stages (compare with Figure 12**a**). **a'**: Frontal view. The white arrows indicate the direction of the epidermal and cuticular overgrowth of the brain region. **a''**: Posterior view. **a'''**: Dorsal view. The white arrows indicate the dorsad growth of tissue that eventually forms the heart (H). **b**: Dorso-lateral view showing the crumbled appearance of the dorsal tissue directly posterior to the head lobes after dorsal closure. The prosomal tergites continue to extend dorsally (white arrows) (compare with Figure 12**a**). **c**: Detail of anterior prosoma. As a result of the forward positioning of the cheliceres and/or posterior positioning of the stomodeum, the labrum (Lb) is now between the bases of the cheliceres (Ch). Cuticular formations (white arrows) are visible in the sternal regions (white asterisks) of the prosomal segments. **d**: The right limb bud on opisthosomal segment two (O2) shows four pulmonary furrows (black arrows) and a lateral opening of the pulmonary sac (PuS). At the latero-posterior insertion of the limb bud at opisthosomal segment three (O3), the opening of the tubular trachea (TrO) is visible. **e**: Posterior opisthosomal region. The anlagen of the left and right tergite plates (Ter) meet dorso-medially (compare with a later stage, Figure 14**c**). en, endite; L1-L4, walking legs one to four; P, pedipalp; VS, ventral sulcus.

Dorsal closure completes inversion. The tergites of both body halves meet along the dorsal midline, covering all of the dorsal yolk with embryonic tissue. This gradual event starts posteriorly with the tergites of the caudal region and progresses anteriorly. The laterally expanding tissue of the prosomal tergites meets the tissue posterior to the precheliceral lobes (white arrows; Figure 13b). During the process, the dorsal prosomal surface has a crumpled appearance (Figure 13b). Underneath the area where the tergite plates touch each other, tissue that eventually forms the heart becomes evident (Figure 13a'''). Cuticle covers the sternites in the prosoma (white arrows; Figures 13c) and the brain region is also overgrown by epidermal and cuticular formations (white arrow; Figure 13a'). The embryonic tissue anterior to the pedipalp has bent posteriorly, marking the start of the process in which the supraoesophageal area folds onto the suboesophageal area. As a result of the positioning of the cheliceres and stomodeum, the labrum is positioned between the bases of the cheliceres (Figures 13a'. c). The pedipalps and walking legs have further extended and meet each other medially in a zipper-like manner. The posterior sides of the limb buds on opisthosomal segment two have become concave (Figure 13d). Evident on each bud are four pulmonary furrows (Figure 13d; black arrows) and the opening of the pulmonary sack (Figure 13d; white arrow). The segments posterior to opisthosomal segment eight have become compressed, giving them a swollen appearance (Figure 13e).

#### Stage 18, Prosomal shield

**Figure 14:**
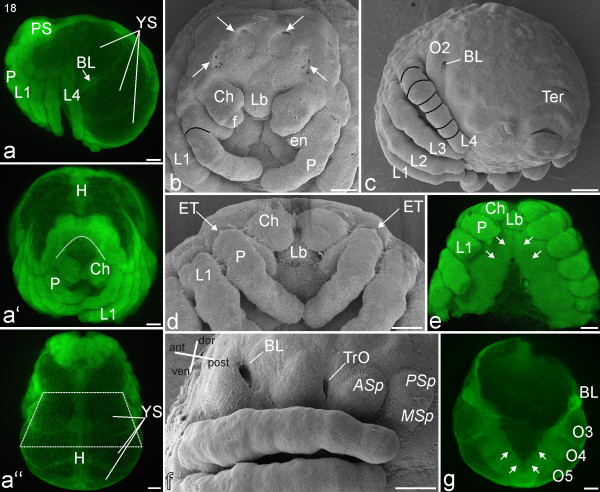
**Stage 18, Prosomal shield**. All scale bars 100 μm. Sytox staining, **a-a''**, **e**, **g**; SEMs, **b-d**, **f**. **a**: Lateral view. The dorsal yolky mass is divided into three distinct yolk sacs (YS). **a'**: Frontal view. The dotted white line shows the position of the labrum posterior to the cheliceres, which partially cover it with their bases. **a''**: Dorsal view. The top of the white (dotted line) trapezoid designates a narrowing region that will eventually become the petiolus, a short length of thin connecting tissue between the prosoma and opisthosoma as shown in Figure 18**g **and 19**b**. The base of the trapezoid indicates the broader opisthosoma that continues in advanced stages (e.g. Figure 18**g**, 19**a**). The heart primordium (H) is evident in the dorsal midline. **b**: Frontal view. The anterior brain region is almost covered by the prosomal shield (white arrows). **c**: Postero-lateral view. The elongated walking legs (L1-L4) show their final segmentation into seven podomeres (black lines). The second opisthosomal segment (O2) shows a prominent opening for the book lung system (BL). **d**: Tiny egg teeth (ET) appear laterally on the most proximal segment of the pedipalps (P). **e**: Ventral view of the prosoma, with the legs trimmed. The sternites start to fuse medially from anterior to posterior (white arrows) in a process that will eventually result in a single sternal plate. **f**: Lateral view of the left opisthosomal region in a late stage 18. The broad openings (primordial spiracles) of the book lung system (BL) and the tracheal (TrO) systems are clearly visible. **g**: Ventral view of the opisthosoma; the prosoma is cut off (same embryo as in **e**). The opisthosomal sternites start to fuse medially from posterior to anterior (white arrows). *Asp*, anlage of anterior spinneret; Ch, chelicere; en, endite; f, fang; Lb, labrum; *MSp*, anlage of medial spinneret; O3-O5, opisthosomal segments three to five; PS, prosomal shield; *PSp*, anlage of posterior spinneret; Ter, tergite.

The rim of the precheliceral lobes grows in the direction of the mouth opening and covers the brain, which has thickened substantially (Figures 14a, a'', b). The *lateral *and *medial subdivisions *are the last parts of the brain to be overgrown. Dorso-posteriorly to the precheliceral region, cuticle continues to expand marking the start of the formation of the prosomal shield (white arrows; Figure 14b). The labrum is now posterior to the cheliceres, which in frontal view partially cover the labrum with their bases (Figures 14a', b, e). Tiny egg teeth appear laterally on the most proximal segment of the pedipalps (ET; Figure 14d). The walking legs are more slender than before and show their final segmentation into seven podomeres (black lines; Figure 14c).

The dorsal yolky mass is divided into at least three distinct yolk sacs (YS; Figures 14a, a''). In parallel, yolk moves from the prosomal segments into the posterior part of the embryo, while simultaneously the petiolus starts to constrict, causing the embryo to lose its spherical shape. Together, these events mark the start of the division into what will later become the tagmata (prosoma and opisthosoma). The opisthosoma has grown in relation to the rest of the embryo: at this stage the width of the petiolus is about 60-70% of the width of the opisthosoma (trapezoid line; Figure 14a'').

Opisthosomal segments two, three and four broaden substantially, especially at their dorsal ends, giving the embryo a crooked appearance (Figures 14a, c). The third opisthosomal segment widens ventrally, with the result that the distance between the book lung primordia and the tracheal tubercles increases (Figure 14g). Ventral closure initiates: The anlagen of the prosomal sternites start to close from anterior to posterior in a process that will eventually result in a single sternum plate (white arrows; Figure 14e). The sternites posterior to opisthosomal segment five have also moved closer together (white arrows; Figure 14g). The most posterior opisthosomal segments, probably segments nine to twelve, have swollen up even more ventrally and form a square-shaped protrusion (Figures 14a, c, g).

#### Stage 19, Heart

**Figure 15:**
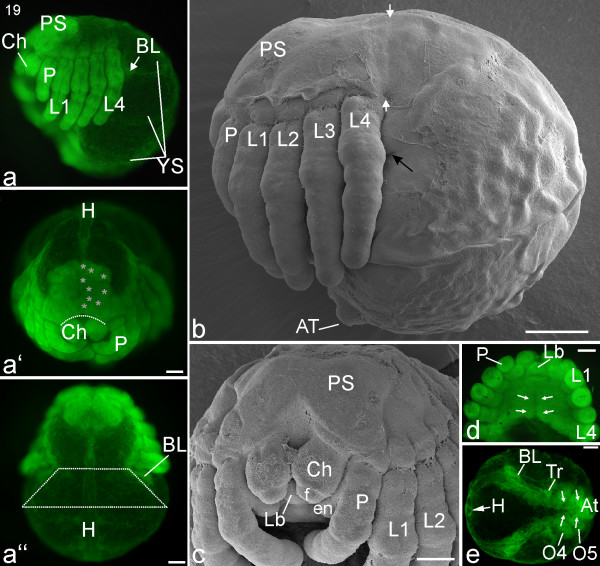
**Stage 19, Heart**. All scale bars 200 μm. Sytox staining, **a-a''**, **d**, **e**; SEMs, **b**, **c**. **a**: Lateral view. BL indicates an internal mass of cells that will become book lung tissue. The brain region has sunk into the prosoma (compare with earlier stages, e.g. Figure 18a) and is partially covered by the prosomal shield (PS). **a'**: Frontal view. The dotted line indicates the advancing edge of the prosomal shield that will eventually cover the brain region. The grey asterisks (on the left half of the body only) show that the brain has differentiated into interconnected lobes. **a''**: Dorsal view. As described in the legend for Figure 14, the top of the trapezoid (white dotted lines) spans the narrowing region that will become the petiolus while the base of the trapezoid shows the continuing breadth of the opisthosoma. The tubular heart (H) is developing in the dorsal midline and is the main identifying feature for this stage. **b**: Lateral view. A suture between the prosoma and opisthosoma is visible (white arrow heads). Some embryonic cuticle was torn off, exposing the opening of the book lung system (black arrow). The posterior-most segments of the opisthosoma are further compressed and together form the anal tubercle (AT). **c**: Frontal view. The brain region is almost completely covered by the prosomal shield (PS) and the labrum (Lb) lies ventral to the cheliceres (Ch). **d**: Ventral view of the prosoma. The legs are trimmed to show the medially fused sternites (indicated by white arrows) of all prosomal segments. **e**: Ventral view of an opisthosoma separated from the prosoma (same embryo as **d**). White arrows indicate the progress of the ventral closure of opisthosomal sternites. The tubular heart (H) is seen in cross section as a circular structure. BL, book lung system; en, endite; f, fang; L1-L4, walking legs one to four; O4, O5, opisthosomal segments four and five; P, pedipalp; Tr, tracheal system.

The protocerebral part of the brain no longer sits on the yolk but has sunk into the prosoma (Figure 15a). The prosomal shield has almost completely covered the brain, save for a wedge-shaped opening directly dorsal to the cheliceres (white dotted line; Figure 15a'). In frontal view, the labrum is now fully covered by the cheliceres (Figures 15a', c). The labium has started to protrude, and together with the labrum forms a beak-like structure. Posterior to the mouth, the prosomal sternites have fully closed (white arrows; Figure 15d). The prosomal tergites are dorso-ventrally reduced, and the inserts of the pedipalps and walking legs have moved dorsally (Figures 15a, a'', b). From a lateral perspective, the brain and the inserts of the walking legs no longer form a continuous arch but lie at an acute angle to each other. All in all, the prosoma has become more compact, a process probably also accompanied by further movement of yolk from the prosoma into the opisthosoma.

The petiolus has constricted further, and at this stage is about 50-60% of the width of the opisthosoma (trapezoid line; Figure 15a''). Dorsally on the opisthosoma, nuclear staining shows a tubular heart (Figures 15a'', e). Ventrally, the opisthosomal sternites have not yet fully closed. Due to a broadening of the sternite of opisthosomal segment three, the book lung primordia have migrated anteriorly and are now almost completely lateral to the petiolus (Figure 15a''). The posterior-most segments of the opisthosoma have further compressed and together form the anal tubercle (Figure 15b).

#### Stage 20, Ventral closure

**Figure 16:**
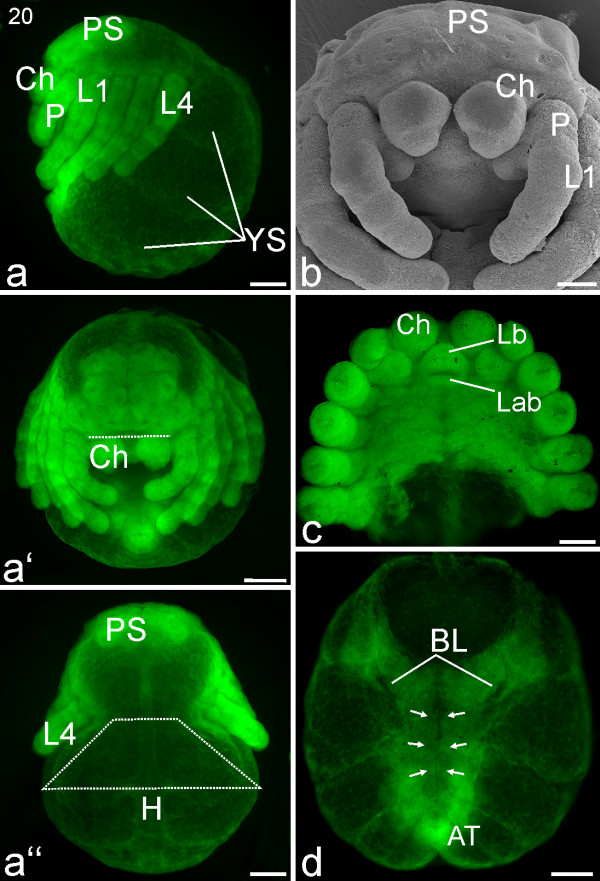
**Stage 20, Ventral closure**. All scale bars 200 μm. Sytox staining, **a-a''**, **c**, **d **SEM **b**. **a**: Lateral view. Three yolk sacs (YS) are visible in the opisthosomal region. The brain region is fully covered by the prosomal shield (PS). **a'**: Frontal view. The dotted line indicates the advancing edge of the prosomal shield that at this point fully covers the brain region. **a''**: Dorsal view. As described in the legend for Figure 14, the top of the trapezoid (white dotted lines) spans the narrowing region that will become the petiolus while the base of the trapezoid shows the continuing breadth of the opisthosoma (compare with Figures 14**a''**, 15**a''**). **b**: Fronto-ventral view of the prosoma. **c**: Ventral view of the prosoma, legs are trimmed. **d**: Ventral view of the opisthosoma. The prosoma is cut off so the medial growth of the sternites (arrows) can be seen in the process of ventral closure (same embryo as **b**). AT, anal tubercle; BL, book lung system; Ch, chelicere; H, heart; L1-L4, walking legs 1-4; Lab, labium; Lb, labrum; P, pedipalp; YS, yolk sac.

Nuclear staining of the developing brain shows distinct regions that correspond to brain parts such as the optic ganglia (Figure 16a). The prosomal shield covers the whole prosoma, including the brain region directly dorsal to the labrum (Figures 16a, a', b). The cuticular border of the prosomal shield is now visible between the prosoma and the opisthosoma (Figure 16b). In lateral view, the insertion sites of the walking appendages lie in a straight line (Figure 16a). The width of the petiolus is about 40-50% of the width of the opisthosoma (trapezoid line; Figure 16a''). The embryo has become even more crooked, such that the first pair of walking legs almost touches the anal tubercle (Figures 16a, a'). Ventral closure of the opisthosoma is complete, and the book lungs have moved antero-medially in the direction of the petiolus (Figure 16d). The spinnerets of both body halves lie close together and form the spinning field. The spinning field has moved close to the anal tubercle (AT; Figures 16a', d).

#### Stage 21, Petiolus

**Figure 17:**
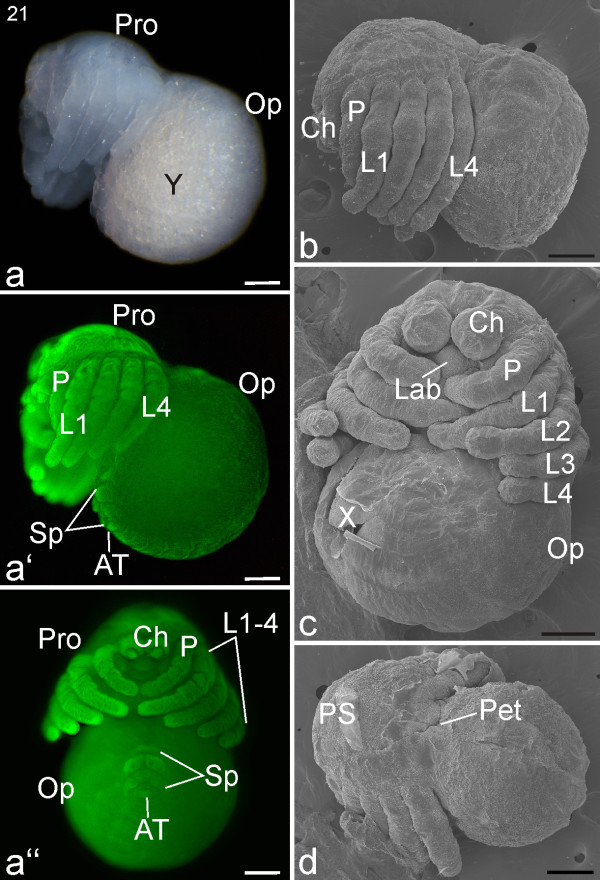
**Stage 21, Petiolus**. All scale bars 200 μm. Light micrograph, **a**; Sytox staining, **a'**, **a''**; SEMs, **b-d**. **a**: Lateral view. A narrowing region (petiolus) connects the prosoma (Pro) and opisthosoma (Op). The colour of the opisthosoma is distinct from the prosoma due to the large proportion of yolk (Y) **a'**: Lateral view of same embryo as in **a**. The prosomal shield (PS) is clearly evident, as are the differentiating spinnerets (Sp). **a''**: Ventral view, same embryo as in **a **and **a'**. The lobes of the spinnerets (Sp) and anal tubercle (AT) are prominent. **b**: Lateral view. **c**: Ventral view. **d**: Dorsal view. The SEMs of **b**, **c **and **d **show that the swollen opisthosoma is connected to the prosoma by a narrowing petiolus (Pet). The appendages are long and segmented, and the prosomal shield (PS) is prominent. Due to a cuticle cover (torn edges evident in **c**), the opisthosomal surface shows little indication in SEM images of the spinnerets that are evident with Sytox staining in **a''**. Ch, chelicere; L1-L4, walking legs one to four; X, damaged area, cuticle torn.

**Figure 18:**
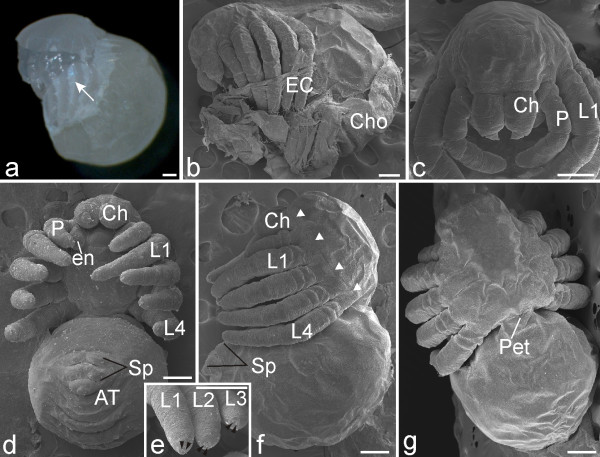
**Hatching and Postembryo of *C. salei***. All scale bars 200 μm. Light micrograph, **a**; SEMs, **b-g**. **a**: Lateral view of a live hatching embryo. White arrow points toward air underneath the egg membranes. This air space between the membranes and the embryo surface becomes evident shortly before hatching. **b**: Lateral view of hatching embryo with chorion (Cho) and embryonic cuticle (EC) partially removed. **c**: Frontal view of newly hatched postembryo. **d**: Ventral view of postembryo. The spinneret anlage (Sp) is now clearly evident as bilateral bulges in the ventral opisthosoma. **e**: Detail of the tips of the walking legs of the postembryo. Black arrowheads indicate tarsal claws. **f**: Lateral view. White arrowheads mark the line where the cuticle of the postembryo will eventually break for the first moult to first instar. **g**: Dorsal view. The narrow petiolus (Pet) connects the prosoma and opisthosoma. AT, anal tubercle; Ch: chelicere; en, endite; L1-L4, walking legs one to four; P, pedipalp.

In the course of this stage, a complete cuticle develops underneath the embryonic cuticle, making it impossible to obtain information about the inner morphology using nuclear staining of whole mounts. Externally, the fangs of the cheliceres become pointed and are directed towards each other (Figures 17a'', c). The final restriction of the petiolus takes place; the embryo starts unfolding and loses its crooked appearance (Figures 17a-d). Seitz [26] observed in advanced embryos (probably corresponding to stage 21) that lateral parts of the prosoma had not yet been fully covered by the dorsal shield. We cannot confirm this however. Towards the end of this stage, air appears between the prosomal appendages, indicating that the embryo is taking up the exuvial liquid which will allow it to exert pressure on the egg membranes (white arrow; Figure 18a).

### Postembryonic stages

#### Postembryo

Eclosion marks the end of the embryonic stages. In *C. salei*, this process includes the rupturing of the egg membranes and moulting from the embryonic cuticle (EC; Figure 18b). The rupturing of the egg membranes invariably starts around the pedipalps, and is likely initiated by the pressure of the egg teeth on the membranes. The embryonic cuticle, which bears the egg teeth, also opens along a predetermined breaking line around the carapace (Figures 18a, b). The resulting stage, which we name the 'postembryo' after [47] is completely immobile. The outer appearance is very similar to late embryonic stage 21, with legs that still bend ventrally. However, once released from the egg membranes, the postembryo is completely unfolded. The spinnerets and anal tubercle become more pronounced (Figures 18c-g) and the first pigments can be seen in the eyes. The pointed fangs have not yet fully extended (Figure 18c) and the endite of the pedipalps does not touch the other mouth parts (Figure 18d). The postembryo has two tiny tarsal claws on the tip of each leg (black arrow heads; Figure 18e). No sensory hairs are visible on the cuticle.

#### First instar

**Figure 19:**
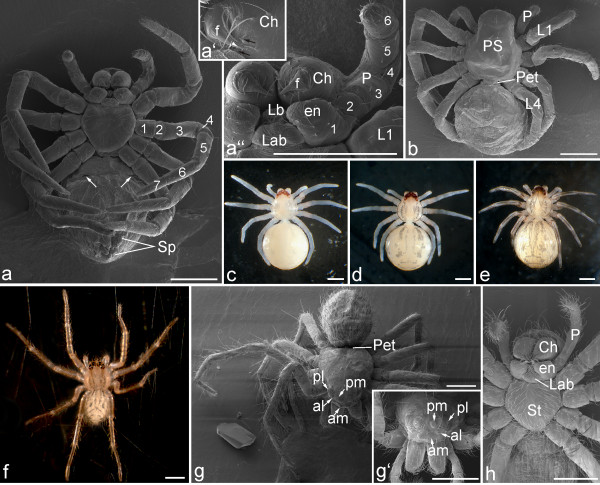
**First and Second instar of *C. salei***. All scale bars 500 μm. SEMs, **a-b**, **g-h**; light micrographs, **c-f**. **a-e First instar**. **a**: Ventral view. Numbers indicate the seven podomeres of L2. White arrows indicate the slit-like opening of the book lung system. **a'**: Detail of the tip of the left chelicere showing the fang (f), a sharp cuticular claw on which the opening of the poison gland is visible (white arrow head). Black arrows indicate the retromarginal teeth at the base of the chelicere. **a''**: Detail of the mouth region. Numbers indicate the six podomeres of the pedipalp. **b**: Dorsal view. **c-e**: Dorsal view of increasingly older first instars, illustrating the increase in pigmentation. **f-h Second instar**. **f**: Dorsal view. **g**: SEM image. Dorsal view shows the arrangement of the eyes (al: anterior lateral eye, am: anterior median eye, pl: posterior lateral eye, pm: posterior median eye) and the petiolus (white arrow). **g' **Frontal view. Higher magnification of the eye region of **g**. **h**: Ventral view. The mouth opening is surrounded antero-laterally by the two cheliceres (Ch), laterally by the endites (en) of the pedipalps (P) and posteriorly by the labium (Lab). The labrum anterior to the mouth opening is covered by the cheliceres. L1-L4, walking leg one to four; Lb, labrum; Pet: petiolus; PS, prosomal shield; Sp, spinnerets; St, sternum.

The first instar emerges from the postembryo after about 3 days (at 25 C). Contrary to earlier observations [48] we never witnessed a first instar hatching directly from the egg. The walking legs of the first instar extend laterally (Figures 19b, c-e). The cheliceres have two so-called retromarginal teeth on their bases (black arrows; Figure 19a'). Both teeth are positioned opposite the folded fangs. Distal-laterally, the fangs bear an opening to the poison gland (white arrow head; Figure 19a').

Although the first instar cannot walk, it bears sensory hairs on the cuticle and reacts to tactile stimuli by wiggling its appendages (Figures 19a, b). The opisthosoma is spherical in shape, still contains yolk reserves, and is about twice as big as the prosoma (Figures 19b-e). The first instar probably obtains nutrients mainly from this yolk reserve, but animals were frequently observed feeding on unfertilized eggs as well.

The cuticle of the first instar is transparent and not pigmented. After about 10 days an increase in cuticle pigmentation can clearly be seen (Figures 19c-e). In addition, the pigmentation of the eyes becomes more intense. The first silk is produced from the spinnerets, as evidenced by the fine threads that hold the first instars together when the cocoon is opened at this stage.

#### Second instar

The prosoma has not grown substantially from the first instar: the opisthosoma has reduced drastically and is only slightly larger than the prosoma (Figures 19f, g). The second instar may have little or no yolk reserves, as suggested from the reduced size of the opisthosoma (Figure 19f). The walking legs have doubled in length, coinciding with the ability to walk. The cuticle is pigmented, with a grey striped pattern on a light brown background (Figure 19f). At this stage, the mother opens the cocoon and the young spiders leave it to forage and disperse [7].

## Discussion

### The germ disc and the equator

As noted by previous authors [5,26]*C. salei *is significantly different from many other spider species because no dense germ disc is formed during its development. A germ disc (a circular aggregation of blastodermic cells) represents the first visible formation of embryonic tissue and is found in many arthropod groups (e.g. onychophorans [49], myriapods [50], crustaceans [51] and insects [52]). Germ discs are usually made up of cells that are almost free of yolk compartments and arranged in a more compact way than the remaining cells. A consequence of germ disc formation is that there are relatively few blastodermic cells in the remainder of the egg; the extra-embryonic region. In many spiders, including *A. tepidariorum*, the germ disc is very dense in comparison with the extra-embryonic part of the egg. It takes up roughly half of the egg, and a pronounced border with the extra-embryonic region circumscribes the egg more or less half-way between the poles [e.g. [5]]. In *C. salei *however, no aggregated cells are noticeable at the point when gastrulation starts with the formation of the blastopore (stage 3, Figures 2h-j: for comparison see stage 3 in [25]). Instead, the blastodermic cells appear to remain evenly distributed over the egg surface and a relatively large amount of yolk surrounds the periplasm. We cannot conclude from our data that no germ disc forms, but if an aggregation of cells does occur around the blastopore, it is very subtle and no clear border is visible during gastrulation (stages 3, 4 and the first half of stage 5). We therefore describe *C. salei *as not having a dense germ disc.

After gastrulation (end of stage 5) in *C. salei *a visible border appears that circumscribes the egg (Figure 3a). Seitz refers to this border between the embryonic and extra-embryonic portions of the egg as the 'equator', and accurately interprets it as the anterior border of the embryo [26]. However, the way in which this structure develops was unclear at the time of publication, as well as how it relates to the germ disc in other spider species. One hypothesis that has been presented is that the equator becomes visible because of an increase in cell density in the embryonic half of the *C. salei *egg, due to ongoing cell division during stage 5 [5].

Our live-embryo movies provide further insight into the nature of the equator, because the lack of a non-transparent germ disc in *C. salei *facilitates the visualisation of cell movements underneath the surface (ectoderm). Shortly before and during the instant when the cumulus separates from the primary thickening, blastodermic cells move towards the blastopore. Simultaneously with this radial movement of the cumulus, additional cells (also from the primary thickening) disperse radially underneath the ectoderm (e.g. following movie frame 560 of Additional file 3). When the cumulus finishes its migration of about 90 degrees over the yolk mass (end of stage 5) these radially migrating cells are distributed over the embryonic hemisphere. This multi-layered hemisphere appears to be more dense than the extra-embryonic hemisphere (Figure 3e; end of movie of Additional file 2). We interpret these uniformly distributed cells as mesendodermal (mesoderm and endoderm) and it is likely that most of them were internalised during formation of the primary thickening. This data thus shows that cell migration from the primary thickening (end of stage 5) rather than cell division in the blastoderm is responsible for the formation of the equator.

The question then arises how the mode of gastrulation of *C. salei *relates to that observed in spider species that do form a dense germ disc [e.g. [32,33,36,53]]. Sections and time-lapse movies of an example of the latter category, *Zygiella x-notata*, showed similar cell movements: 1) The cumulus migrates away from the blastopore; 2) additional cells underneath the ectoderm spread over the embryonic primordium from the blastopore and 3) ectodermal cells move towards the blastopore [32]. Since our data for *C. salei *is similar to the data for *Z. x-notata*, we propose that both species with and without a dense germ disc follow what has been called the 'canonical mode of gastrulation' [32] in that all mesendodermal cells have their origin in the region of the blastopore/primary thickening. Furthermore, we propose that in species with a dense germ disc, a border is also formed that for some as yet undiscovered reason the migrating mesendoderm does not pass. In those species, such a structural equivalent of the equator of *C. salei *is masked by the edge of the germ disc and therefore not readily visible.

These proposals are in discord with one interpretation of gastrulation that has been proposed for *A. tepidariorum *[54,55]. In these publications, the 'rim of the germ disc' is given a prominent organising role. To achieve this, expression of a *twist *homolog (*At-twist) *is used as a molecular marker of spider mesoderm. Expression of *At-twist *is shown in embryos fixed towards the end of stage 5, in cells underneath the blastoderm at the border of the germ disc. Next, in embryos fixed at stage 6, *At-twist *expressing cells are shown distributed over the early germ band. Combining these patterns, the authors conclude that *At-twist *expressing cells internalise at the rim of the germ disc and subsequently spread over the embryo. This data is taken as evidence that the border of the germ disc is an additional site of gastrulation [54] giving rise to so-called 'peripheral mesoderm'. This would imply that *A. tepidariorum *differs from the canonical mode of spider gastrulation.

We argue against this hypothesis. First, similarly to what we show for *C. salei *(Additional files 2 and 3), also in *A. tepidariorum *we have observed centrifugal movements of individual subsurface cells coming from the centre of the germ disc in a time-lapse movie of live embryos (unpublished data, CW). In *C. salei*, cells originating from the blastopore have reached the equator towards the end of stage 5, and it is plausible that this is also true for late stage 5 embryos of *A. tepidariorum*. Second, it cannot be concluded from gene expression in fixed embryos alone that the putative 'peripheral' mesoderm in *A. tepidariorum *internalises at the rim of the germ disc [54] and that it does not derive from the centre of the germ disc. Third, the putative spreading of *At-twist *expressing cells at stage 6 [54,55] is clearly a later event than the centrifugal movement that we observe during stage 5. This spreading therefore does not necessarily depend on internalisation of cells at the rim of the germ disc and does not contradict the canonical view of gastrulation.

Concluding, our data provide an explanation for the appearance of an equator in the absence of a dense germ disc in *C. salei*. Furthermore, we propose that in *C. salei *all mesendodermal cells have their origin in the region of the blastopore/primary thickening, and that this also holds true for species with a dense germ disc such as *Z. x-notata *and *A. tepidariorum*. Consequently we propose that the equivalent of an equator also forms in species with a dense germ disc, and that the nature of this border deserves further attention. For example, which factors prevent mesendoderm from migrating past the equator in *C. salei*? How do these factors relate to those that define the edge of the germ disc in *A. tepidariorum*? These questions show that a combined study of more than one species is necessary for a better understanding of the early development of spiders. Also, from a broader perspective, the variation in density of the germ disc in spider embryos provides evidence for the idea that early developmental stages can be modified during evolution without affecting later stages, and that homologous developmental stages are not necessarily based on homologous earlier stages [56].

### Brain development

The first signs of neurogenesis can be observed in stage 11 of *C. salei *once gastrulation has taken place, the a/p axis has formed and the germ band has started to elongate. Evenly dispersed point-like depressions appear on the precheliceral lobes and ventral neuroectoderm (Figures 7a', a'', b). These depressions are caused by the immigration of cell groups and are also called 'invagination sites' [e.g. [57]]). In addition, depressions are visible on the stomodeal anlage (see below). The internalised cells detach from the embryonic ectoderm and develop directly into neural tissue [57].

Differentiation of the brain starts with the formation of the *lateral furrows *in stage 12 (Figures 8a', b). On each precheliceral lobe, a lateral field of neural primordia cells folds inwards and differentiates into the lateral eyes (anterior lateral and posterior lateral eyes, Figures 19g, g'). These bilateral structures have been termed lateral vesicles or invaginations in other arachnids and have been identified as the primordia of the lateral eyes [e.g. [27,39]]. As development progresses and the cells of the *lateral furrow *continue to immigrate (stages 14-16) a medially adjacent cluster of cells - the *lateral subdivision *- begins to cover the *lateral furrow *completely (*ls; *Figures 14b, 16b). This neural primordial tissue differentiates as development progresses into the median eyes (anterior median and posterior median eyes; Figures 19g, g' and [27]).

Simultaneously, a large field of cells at the anterior rim of each precheliceral lobe folds inward to form a characteristically crescent-like shape - the *anterior furrow *(AF; stage 14, Figure 10b). This is the most prominent structure seen during early chelicerate brain development and is often described as the semi-lunar or cerebral groove [e.g. [33,45]]. The invaginating cluster of cells that results in the *anterior furrow *is the neural primordium of the arcuate body. In a procedure similar to that described for the migration of the *lateral subdivision *(median eyes), a *medial subdivision *of neuroectodermal cells starts to overgrow the *anterior furrow *(stages 15-17). The *medial subdivision *fills the space occupied by the *anterior furrow *and represents the neural primordium of the mushroom bodies (Figure 12b, figure 3 in [27]). The now separate brain compartments continue to differentiate and are eventually overgrown by a hood (Figures 14b, 15c, stages 18-19, see [27]). The tissue for the hood comes from the outer rim of the precheliceral lobes and encloses not only the entire brain complex but also the dorsal prosomal region to form what is known as the prosomal shield. At the same time, an extreme backwards bending of the precheliceral region during the process of inversion brings the brain compartments into their final position.

For the insights they provide about the sequence of early brain differentiation, our data complement earlier findings [27] regarding the three dimensional arrangement of brain parts such as the arcuate body, the mushroom body and the optic ganglia. Our data provide information about the area from which these primordial tissues originate. We are able to map the precursor cells of single brain regions (arcuate body, mushroom body and optic ganglia) onto the two dimensional tissue layer of the precheliceral neuroectoderm. As development progresses, the neural tissue undergoes complete spatial reorganization and differentiates into the mature brain via complex morphogenetic processes

### Mouth region

The mouth develops in close association with the developing brain. The first depression of the stomodeal anlage appears in the course of stage 11 between the precheliceral lobes (Figure 7b). By stage 12 the labrum (upper lip) appears as a bi-lobed structure directly anterior to the stomodeal anlage, which by now has subsided somewhat (Figure 8b). As development progresses, the mouth opening gradually migrates posteriorly, while in the course of stage 14 the labium (lower lip) appears and the lobes of the labrum fuse (Figure 10b). The result of the posterior migration of the mouth opening is an increasingly pronounced cleft between the two precheliceral lobes (visible for example in Figure 12b). After the first true moult into the first instar, the endites of the pedipalps (also known as the maxillae) form the lateral border of the mouth opening (Figure 19a').

The early development of the mouth region of chelicerates has been the subject of recent publications [37,40]. Three small point-like invaginations were observed in the centre of the stomodeal anlage of the spider *L. geometricus *[37]. Since the proboscis of pycnogonids (sea spiders) has a tri-radial construction and this tri-radial symmetry is established during the formation of the stomodeum [40,58,59], the occurrence of a triangular stomodeal invagination has been used as an argument for a sister group relationship between pycnogonids and euchelicerates (vs. the Cormogonida hypothesis, according to which the sister group relationship is between pycnogonids and extant euarthropods) [59]. In *C. salei *at stage 11 (Figure 6b) and in *A. tepidariorum *(unpublished data, CW) we have observed three invaginations in the stomodeal anlage that we also interpret as the result of internalised neural precursor cells. Due to ongoing mouth formation, these invaginations are no longer externally visible by stage 14 (Figure 10b). Previously [27] a crescent-shaped array of neural primordial cells has been described in the dorsal part of the stomodeum at 220 hAEL of *C. salei *development (comparable to stage 15; see Figure 1). The authors assume that these invaginating neural precursors give rise to the stomatogastric nervous system, which controls the motions of the gut. To test whether the triangular pattern is a valuable character for making phylogenetic inferences, we need to know more about the neural structures that result from these particular neural precursor cells. A Y-shaped stomodeal invagination was also found in the isopod crustacean *P. scaber *[24] for example, indicating that this structure may actually represent an arthropod plesiomorphy (ancestral character) rather than a synapomorphy of any particular arthropod subgroup. With the techniques available for *C. salei*, the species would be an appropriate starting point for such investigations, especially in light of the fact that its central and peripheral nervous system has been the focus of previous studies [27,48].

The nature and evolutionary origin of the labrum is another open question relating to the mouth region in arthropod evo-devo studies [e.g. [60]]. It is well documented that the spider labrum develops from a bi-lobed structure (also known as the 'rostral appendages') that fuses into a single structure (or 'rostrum') [33,36,37,61]. We observed the same sequence in *C. salei; *two bulges first appear at stage 12 (Figures 8a', 8b), become more pronounced during stages 13 and 14 (Figures 9a', 9b, 10a', 10b) and fuse at stage 15 (Figures 11a', 12a', 12b) to form the labrum. Additionally, gene expression studies show that several leg patterning genes are expressed in the labrum in an appendage-like way [62]. These data support the argument that the labrum consists of appendages from the protocerebral region (ocular segment) that fuse in the course of embryonic development [60].

The bi-lobed nature of the labrum is also described in a recent study of *L. geometricus *[37]. Furthermore, it has been observed that before the first appearance of the labrum, a distinct and slightly depressed field appears between the two precheliceral lobes which the authors refer to as the 'mouth region' [37]. The authors hypothesise that it is within this mouth region that the stomodeal opening forms and the two labral bulges (which the authors call 'hypostome') develop [37]. We do not observe a distinct field of this nature between the precheliceral lobes in *C. salei *(Figures 6 and 7). Moreover, the raw SEM data presented for *L. geometricus *(Figure 3b in [37]) is not entirely clean, and the impression of a depression might derive from shrinkage during fixation.

### Inversion and the post-opisthosoma

As described in the Results section, inversion in *C. salei *involves a drastic rearrangement of different parts of the body due to simultaneous migration and expansion of tissue over the yolk mass (Figures 10, 11, 12 and 13). Yolk is incorporated into embryonic tissue, and parts of the brain fold backwards over the ventral nerve cord (Figure 1 in [27]). It is generally agreed that inversion starts with a pronounced increase of the widening of the ventral sulcus [e.g. [30,63]] which corresponds to stage 14 of *C. salei *(Figure 10a''). The precise end, however, is less well defined. A study of the species *Latrodectus mactans *described the end as when "the shift of tissues toward the mid-ventral line is completed" [[36], p.49], which would imply that inversion finishes at ventral closure, i.e. at stage 20 in *C. salei*. Other interpretations suggest that inversion ends when all the yolk is covered by ectoderm [35] or that it finishes with the dorsal closure of the prosoma [30]. Dorsal closure is more practical to observe than ventral closure because for observation of the latter the legs must be removed. Furthermore, during inversion the ventral sulcus consists of a (thin) layer of tissue and thus covers the yolk. We therefore use dorsal closure to define the end of inversion in *C. salei *(stage 17). We then divide the inversion movements preceding dorsal closure into three separate stages (stages 14-16; Figure 11d) based on the progress of the movement of the germ band halves over the yolk mass.

Inversion itself has undergone some significant changes over the course of spider evolution. In some species like the basally branching *Heptathela kimurai*, the ventral sulcus does not widen to incorporate the yolk mass. Instead, most opisthosomal segments develop as a protuberance that flexes ventro-anteriorly, extending from the yolk mass [33]. This so-called post-opisthosoma initially contains very little yolk, but gradually fills up with more yolk from the more anterior segments [33]. It has been proposed that this way of internalising the yolk represents the ancestral state [63]. Other species show an intermediate morphology. In *Segestria bavarica *for example, the ventral sulcus of opisthosomal segments 1 to 3 widens ventrally and the two halves of the germ band move dorsally over the yolk mass, similar to most opisthosomal segments of *C. salei*. The rest of the opisthosomal segments, however, form a forwardly flexed post-opisthosoma [46], similar to in *H. kimurai*. Entelegyne spiders, such as *C. salei *or *A. tepidariorum*, are thought to display the most derived character state [46,63] with a strongly reduced post-opisthosoma. In *C. salei*, the post-opisthosoma consists of the tissue posterior to opisthosomal segment nine only. The tissue lies flat on the tergites of opisthosomal segments seven to nine (Figure 12e). Additional file 4 compares the post-opisthosomas of *S. bavarica *and *C. salei*.

It is unclear why inversion evolved in the first place, and why the post-opisthosoma seems to have become reduced over time. One proposed scenario is that this happened as "an adaptation to a secondarily increased amount of nutritional yolk in the egg" [[64], p.213]. Another possibility is that the evolution of inversion relates to a change in speed of development. In order to try to answer these questions, one could compare the developmental modes of different spider species taking into account factors such as relative yolk content and duration of development. Furthermore, since the molecular basis of spider segmentation and segment specification has been studied intensively in *C. salei *and *A. tepidariorum *[e.g. [65] and references therein] one might investigate factors involved in the opening of the ventral sulcus and the specification of the post-opisthosoma in these species.

### Respiratory system

During inversion, the breathing organs (the book lungs and tracheae) start to differentiate. It is widely accepted that arachnid book lungs are derived from the book gills of an aquatic xiphosuran-like ancestor [30,66,67] and that the tracheal systems found among arachnids are a derived character [41,68]. However, there is no consensus on the homology of the book lungs and the tracheal system. The traditional view [35,36,42] holds the spider tracheal system to be derived from a book lung system (making them homologous structures) while more recent studies question whether tracheal systems really do originate in the book lungs [e.g. [66]]. Purcell [42] proposes that the tracheal system in spiders originates either from a book lung system or from an ectodermal tendon [42] and argues that it thus evolved independently.

Our data contributes to this discussion by showing the progression of development of the book lungs in opisthosomal segment two and the tracheal system in opisthosomal segment three. Development in opisthosomal segment two starts with a conspicuous depression of the cells posterior to the limb buds which later differentiate into the transverse slit-like openings of the book lung system (Figures 7e, 9d). As development progresses, the opening of the pulmonary sac is formed laterally, followed by the addition one by one, medially, of the book lung openings. In opisthosomal segment three, initial depressions form in the course of stage 11 that look similar to those of the book lung primordia (white arrows; Figure 7e). However, by stage 12, these depressions are no longer visible, and the surface of the embryonic tissue is smooth again (Figure 8c). At stage 16 the third book lung opening is formed in opisthosomal segment two. Only at this stage, a new opening of the tracheal system becomes visible in opisthosomal segment three (TrO; Figure 12d). The position of this new opening is posterior to the limb buds of segment three, but slightly more lateral than the initial opening of the book lung system at opisthosomal segment two (compare with stage 11; Figure 7e). We interpret these early depressions on opisthosomal segment three as remnants of an ancient book lung system in this segment. The opening that emerges later, more medially at the posterior border to the limb bud, is a different structure. The observation that the tracheal opening appears much later than the opening of the book lung system in opisthosomal segment two has also been made for *L. mactans *[36]. This supports our hypothesis that the book lungs and the tracheal system have different developmental origins in *C. salei *and are therefore not homologous structures.

Our data also has implications for the interpretation of a more recent study that uses molecular markers to investigate the evolutionary relationship between arthropod respiratory structures. It is shown that in *C. salei*, the gene *pdm/nubbin *is expressed in all four developing opisthosomal limb buds in a similar expression pattern, and from this it is concluded that structures such as book lungs, tracheal systems and spinnerets might represent evolutionary modifications of an ancient gill structure that was a limb branch (the so-called epipod) [10]. However, in *C. salei *it is difficult to understand which parts of the opisthosomal limbs give rise to which part of the respiratory system. Our data show that both respiratory structures (book lung system and tracheal system) have their origin not in a clear limb structure, but obviously posterior to the limb buds. The possible contribution of a limb structure and its surrounding tissue therefore should be re-investigated in more detail at both a morphological and a molecular level before any conclusions can be drawn as to the homology of spider respiratory structures with breathing structures in other arthropod lineages.

Finally, our data also give some indication of the developmental mechanisms that contribute to the generation of the respiratory structures. Our SEM samples often showed cellular debris in the form of small globules in the area of the forming book lung opening (Figure 6e). This could indicate that the book lungs are not only the result of an ectodermal invagination, as observed by some authors [e.g. [67]] but that cell death could also play a role in their formation. The relative contribution of both of these mechanisms must be investigated further if we are to improve our understanding of the development of this complex system and its evolutionary origin.

### Prosomal and opisthosomal sternites

After dorsal closure, the general morphology of the embryo gradually changes from a round shape to a typical spider body plan with two separate tagmata (prosoma and opisthosoma, Figures 14, 15, 16 and 17). This process involves the constriction of the petiolus (see the trapezoids in Figures 14a'', 15a'', 16a'') and movement of yolk from the prosoma into the opisthosoma. In parallel, both the prosoma and opisthosoma close ventrally.

In the prosoma, ventral closure marks the beginning of the formation of a sclerotised plate, the 'sternum', in between the coxae of the walking legs. Few studies deal with the formation of the sternum, and no definitive information is available on the origin of the sternal tissue [33,36,37,69]. For example, it is unclear whether the single cuticular sternum in adults has a segmental origin and if so, what segments are involved [69]. However, it is known that in this region the ventral neuroectoderm is overgrown by an epidermal layer of cells from lateral regions [14,33]. In *C. salei *this takes place when neurogenesis is over, shortly before ventral closure [14]. We have been able to confirm the overgrowth process using phalloidin staining (data not shown). The overgrowth becomes visible for the first time in the course of stage 18. Unfortunately, this epidermal layer lacks morphological features, such as borders of sclerites or muscle attachments. Therefore, this observation does not provide any conclusions regarding the segmental nature of the sternum.

Nevertheless, the ventral neuroectoderm does exhibit pronounced segmental organization when the prosoma starts to close medio-ventrally (stage 18; Figure 13e). We interpret these segmentally iterated structures as anlagen of sternites. During ventral closure (stages 18-20) the sternal units become more substantial and finally merge into the sternum. In the course of this process, newly formed cuticle covers the ventral prosomal tissue, thus masking the segmental appearance of the sternites (in SEM samples, cells underneath a cuticle are typically no longer detectable).

In the opisthosoma, the process of ventral closure is more complex and our data do not show exactly what happens here with regard to the segmental (sternal) compartments. Significant morphogenetic transformations and extensive tissue reduction in the medio-ventral region of this tagma are probably necessary for the successful differentiation of organ systems such as the respiratory system, the genitals, and the spinning apparatus. The second opisthosomal segment widens, while the more posterior segments are reduced. More information about the tissue covering the opisthosomal neuroectoderm would be extremely helpful in enabling us to draw conclusions about the segmental organisation of the opisthosomal region within arachnids [e.g. [70]].

### The post-embryonic stages

Embryonic development finishes with eclosion (Figures 18a, b). The outer appearance of the final embryonic stage (stage 21; Figure 17) is very similar to the first post-embryonic stage. Nonetheless, sections of *C. salei *post-embryos (unpublished data, MH) and late embryonic stages of *L. mactans *[36] show that many internal changes are taking place, including the development of the reproductive system and the silk glands. After eclosion, the peripheral nervous system also continues to develop [48].

Disagreement about nomenclature and lack of consistent usage of nomenclature complicate comparative studies of the late embryonic and post-embryonic stages of spiders [47]. Part of this problem is likely to stem from the fact that there is great variation in the number and nature of stages in different spider species [46]. Instead of taking this variation into account, research papers on single spider species often stage this period of development according to secondary literature that is over-simplified and outdated regarding this subject [41]. In accordance with a comprehensive review of the post-embryonic stages and a proposed standardization of the terminology [47] we have revised the naming of the stages of *C. salei *(Figure 1).

One of the most confusing and yet persistent terms is 'larva' [41,71]. Its use is unfortunate because, like 'nymph' and 'imago' [71] the term larva derives from the terminology of insect holometabolic development, which implies metamorphosis. We therefore reject this term for the staging of *C. salei*. Instead, we name the stage directly following eclosion and simultaneous shedding of the embryonic cuticle 'postembryo' (after [47], see Figure 18). This postembryo is completely immobile and lacks sensory hairs. The stage was previously referred to as: 'second pre-larva' [7] or 'stage 1 larva'[48].

After the next moult, the 'first instar' emerges (Figures 19a-e). This stage was previously identified as 'larva' and 'third incomplete stage' [7] or 'stage 2 larva' [48]. The first instar is followed by the 'second instar' (Figure 19f-h). The second instar was previously called 'first complete stage' [7] or 'stage 3 larva' [48]). Subsequent instars are numbered further until the sexually mature adult emerges from the penultimate instar. Typically, the adult stage is the twelfth instar. When underfed, sexual maturity can be reached as early as the tenth or eleventh instar [5,7]. Such developmental plasticity is not uncommon for spiders [72].

## Conclusions

We describe here the normal development of *C. salei*, and provide an improved staging system. Stages 1 to 9 follow the existing stages of the spider *A. tepidariorum*, thus facilitating comparative studies of early development. For example, we compare our observations of gastrulation of *C. salei *with data available for *A. tepidariorum*, and conclude that in both species all mesendodermal cells have their origin in the region of the blastopore/primary thickening. Our observations of the migration pattern of these mesendodermal cells underneath the ectoderm also provide an explanation for the poorly understood 'equator' of *C. salei*, and we propose that the equivalent of an equator is also formed in species with a dense germ disc, such as *A. tepidariorum*.

Newly defined stages 10 to 21 cover late development. For this period of development in particular, we provide descriptions that are detailed and comprehensive, since late spider development has been under-studied compared to early development. These descriptions, therefore, provide a strong framework for comparative studies of late developing and differentiating organs, such as the brain, the breathing organs and the silk producing system. For example, we provide many details about the differentiation of the precheliceral region, the morphogenetic process of inversion and the development of the respiratory system. We find that the order of appearance of epithelial invaginations provides evidence for the non-homology of the tracheal and book lung respiratory systems.

Finally, for both early and late development, we provide numerous directions for future research. Such research will advance our understanding of spider development, and will tell us more about how spiders evolved to occupy their unique ecological niche.

## Materials and methods

### Live-embryo imaging

Embryos were mounted on microscope slides and covered with 10S Voltalef oil (VWR International). Embryonic development was recorded using an Axiophot2 Image (Zeiss) equipped with software for 4D-recording, according to published methods [73]. The videos were created using the image processing software 'ImageJ' together with a macro file (kindly provided by Kai Lindemann and available on request). The macro automatically recognizes sharp areas from the different levels and combines them to form a single 'focused image', after which the stack of focused images is merged to a movie.

### SEM imaging

Fresh embryos were fixed for at least four hours on a wheel in Dubosq-Brasil fixative. Freshly made working solution consists of 14 parts stock solution and one part glacial acetic acid. The stock solution consists of 60% ethanol, 8% formaldehyde and 0.5% picric acid. The embryos were washed for at least 12 h in 70% ethanol. They were dehydrated in a graded ethanol series and were either critical point dried in CO2 (BALTEC CPD 030) or air-dried after transfer into hexamethyldisilazane (HMDS) by way of an intermediate step in a 1:1 mix of HMDS and absolute ethanol. The dried samples were mounted on stubs and sputter-coated with gold (BALTEC SCD 005). Images were taken on a LEO 1450 VP and arranged in Adobe Illustrator or Corel Draw.

### Fluorescent staining and imaging

Embryos were collected at different stages of development, and fixed according to standard methods [74]. After rehydrating the embryos into PBS supplemented with 0.1% Tween 20, we incubated them for approximately one hour with about one ng/ml of the fluorescent nuclear marker DAPI (Roche) or Sytox Green (Molecular Probes). After several washes they were photographed under a dissecting microscope (Zeiss Lumar) in the z-stack-mode. This allowed us to get a better impression of the three-dimensional composition of embryonic stages. All the pictures in a z-stack were merged in HeliconFocus (Helicon Soft Ltd.). In Adobe Photoshop or Corel Draw, the images were cropped and colour saturation and contrast were optimized.

## Competing interests

The authors declare that they have no competing interests.

## Authors' contributions

MH and CW designed and coordinated the study, participated in its performance and drafted and wrote the manuscript. Both authors read and approved the final manuscript.

## Supplementary Material

Additional file 1 **Tabular overview, comparing the new staging system and nomenclature for the development of *C. salei *with the system and nomenclature used in earlier publications**. hAEL: hours after egg laying, *[26] **[7] ***[48]. Names/annotation of the stages used in earlier publications are provided, along with an English translation: *Frühe Periplasmafelderung - *Early periplasma aggregation; *Kerne - *nuclei; *Späte Furchung - *late cleavage; *(Großzelliges/Frühes) Blastoderm.- *(large-cell/early) blastoderm; *(Späte) Eikontraktion - *(late) egg contraction; *(Frühe/Späte) Primitivplatte (dreischichtig) - *(early/late) primitive plate (three-layered); *(Früher/ Später) Primitivpfropf - *(early/late) primary thickening; *Keimhemisphärenausbildung - *germ field formation; *Keimhemisphäre *- germ field; *Später unsegmentierter Keimstreif - *(late) unsegmented germ band; *Thoraxsegmentierung - *thorax segmentation; *Bildung der Thoraxextremitäten - *development of prosomal appendages; *Abdominale Segmentierung - *opisthosomal segmentation; *Abdominalknospenbildung - *opisthosomal limb bud formation; *Umrollung - *inversion; *Abgeknickter Embryo - *crooked embryo; *Streckung des Embryos - *stretching of the embryo; *Erstes Postembryonalstadium - *first post-embryonic stage; *(1./2.) Prälarva - *(first/second) prelarva; *Jungspinne - *spiderling; *Larve - *larva. Click here for file

Additional file 2 **This movie shows the embryonic development of four embryos at room temperature (about 20 C)**. Embryo 1 is at the top left of the frame, embryo 2 top centre. The time interval between each frame is 6 minutes. The movie starts at early stage 2 with the formation of the blastoderm and ends in frame 980 with the formation of the equator in late stage 5. Click here for file

Additional file 3 **This movie shows the embryonic development of an embryo at room temperature (about 20 C)**. The time interval between each frame is six minutes. The movie starts at stage 2 and shows in particular the formation of the primary thickening (around frame 350) and the migration of the cumulus (beginning at around frame 580) and other mesendodermal cells. Click here for file

Additional file 4 **Comparison of inversion and 'post-opisthosoma' of *C. salei *and *S. bavarica***. Drawings modified from [46], **a-c**; SEMs, **d-f**. Images are false-coloured to show the exposed area of the yolk mass in red and the ventral sulcus in green. **a**: Lateral view of an *S. bavarica *embryo, stage shortly before opisthosomal limb buds appear. The blue dotted line indicates the primordium of the post-opisthosoma. **b**: Lateral view of an *S. bavarica *embryo at the onset of inversion. The blue dotted line indicates the ventrally flexed post-opisthosoma, consisting of opisthosomal segments four to twelve. **c**: Lateral view of an *S. bavarica *embryo shortly before dorsal closure. **d-e**: Lateral view of *C. salei *stages 10 and 14 respectively. Blue dotted line in **e **indicates the primordium of the post-opisthosoma. **f**: Dorso-lateral view of C. salei stage 16. Blue dotted line indicates the dorsally flexed post-opisthosoma, consisting of opisthosomal segments nine to twelve. Click here for file

## References

[B1] Damen WGM Parasegmental organization of the spider embryo implies that the parasegment is an evolutionary conserved entity in arthropod embryogenesis Development 2002 129 1239 1250 11874919 10.1242/dev.129.5.1239

[B2] Regier JC Shultz JW Zwick A Hussey A Ball B Wetzer R Martin JW Cunningham CW Arthropod relationships revealed by phylogenomic analysis of nuclear protein-coding sequences Nature 2010 463 1079 1083 10.1038/nature08742 20147900

[B3] Rota-Stabelli O Campbell L Brinkmann H Edgecombe GD Longhorn SJ Peterson KJ Pisani D Philippe H Telford MJ A congruent solution to arthropod phylogeny: phylogenomics, microRNAs and morphology support monophyletic Mandibulata Proc Roy Soc B: Biol Sci 2011 278 298 306 10.1098/rspb.2010.0590 PMC3013382 20702459

[B4] Jenner RA Wills MA The choice of model organisms in evo-devo Nat Rev Genet 2007 8 311 319 10.1038/nrg2062 17339879

[B5] McGregor AP Hilbrant M Pechmann M Schwager EE Prpic NM Damen WGM *Cupiennius salei *and *Achaearanea tepidariorum*: spider models for investigating evolution and development BioEssays 2008 30 487 498 10.1002/bies.20744 18404731

[B6] Barth FG A Spider's World: Senses and Behaviour 2002 Berlin Springer-Verlag21676461

[B7] Melchers M Zur Biologie und zum Verhalten von *Cupiennius salei *(KEYSERLING), einer amerikanischen Ctenide Zool Jb Syst 1963 91 1 90

[B8] Weihmann T Karner M Full R Blickhan R Jumping kinematics in the wandering spider *Cupiennius salei* J Comp Physiol A: Neuroethol, Sens, Neural Behav Physiol 2010 196 421 438 10.1007/s00359-010-0527-3 20405130

[B9] Damen WGM Hausdorf M Seyfarth EA Tautz D A conserved mode of head segmentation in arthropods revealed by the expression pattern of Hox genes in a spider Proc Natl Acad Sci USA 1998 95 10665 10670 10.1073/pnas.95.18.10665 9724761 PMC27952

[B10] Damen WGM Saridaki T Averof M Diverse adaptations of an ancestral gill: A common evolutionary origin for wings, breathing organs, and spinnerets Curr Biol 2002 12 1711 1716 10.1016/S0960-9822(02)01126-0 12361577

[B11] Prpic NM Damen WGM Expression patterns of leg genes in the mouthparts of the spider *Cupiennius salei *(Chelicerata: Arachnida) Dev Genes Evol 2004 214 296 302 15014991 10.1007/s00427-004-0393-5

[B12] Prpic NM Janssen R Wigand B Klingler M Damen WGM Gene expression in spider appendages reveals reversal of *exd*/*hth *spatial specificity, altered leg gap gene dynamics, and suggests divergent distal morphogen signalling Dev Biol 2003 264 119 140 10.1016/j.ydbio.2003.08.002 14623236

[B13] Schoppmeier M Damen WGM Double stranded RNA interference in the spider *Cupiennius salei*: the role of *Distal-less *is evolutionary conserved in arthropod appendage formation Dev Genes Evol 2001 211 76 82 10.1007/s004270000121 11455417

[B14] Stollewerk A Recruitment of cell groups through Delta/Notch signalling during spider neurogenesis Development 2002 129 5339 5348 10.1242/dev.00109 12403706

[B15] Stollewerk A Schoppmeier M Damen WGM Involvement of *notch *and *delta *genes in spider segmentation Nature 2003 423 863 865 10.1038/nature01682 12815430

[B16] Prpic NM Schoppmeier M Damen WGM Gene silencing via embryonic RNAi in spider embryos Cold Spring Harb Protoc 2008 10.1101/pdb.prot5070 21356701

[B17] Prpic NM Schoppmeier M Damen WGM Whole-mount In situ hybridization of spider embryos Cold Spring Harb Protoc 2008 10.1101/pdb.prot5068 21356699

[B18] Morewood W Hoover K Sellmer J Predation by *Achaearanea tepidariorum *(Araneae: Theridiidae) on *Anoplophora glabripennis *(Coleoptera: Cerambycidae) Great Lakes Entomol 2003 36 31 34

[B19] Hajer J Hrubá L Wrap attack of the spider *Achaearanea tepidariorum *(Araneae: Theridiidae) by preying on mealybugs *Planococcus citri *(Homoptera: Pseudococcidae) J Ethol 2007 25 9 20 10.1007/s10164-006-0198-2

[B20] Moon MJ An JS Microstructure of the silk apparatus of the comb-footed spider, *Achaearanea tepidariorum *(Araneae: Theridiidae) Entomol Res 2006 36 56 63 10.1111/j.1748-5967.2006.00010.x

[B21] Campos-Ortega JA Hartenstein V The embryonic development of Drosophila melanogaster 1997 Berlin: Springer

[B22] Hartenstein V Atlas of Drosophila development 1993 Cold Spring Harbor: Cold Spring Harbor Laboratory Press

[B23] Browne WE Price AL Gerberding M Patel NH Stages of embryonic development in the amphipod crustacean, *Parhyale hawaiensis* Genesis 2005 42 124 149 10.1002/gene.20145 15986449

[B24] Wolff C The embryonic development of the malacostracan crustacean *Porcellio scaber *(Isopoda, Oniscidea) Dev Genes Evol 2009 219 545 564 10.1007/s00427-010-0316-6 20111872

[B25] Akiyama-Oda Y Oda H Early patterning of the spider embryo: a cluster of mesenchymal cells at the cumulus produces Dpp signals received by germ disc epithelial cells Development 2003 130 1735 1747 10.1242/dev.00390 12642480

[B26] Seitz KA Normale Entwicklung des Arachniden-Embryos *Cupiennus salei *Keyserling und seine Regulationsbefähigung nach Röntgenbestrahlungen Zool Jb Anat 1966 83 327 447

[B27] Doeffinger C Hartenstein V Stollewerk A Compartmentalisation of the precheliceral neuroectoderm in the spider *Cupiennius salei*: Development of the arcuate body, the optic ganglia and the mushroom body J Comp Neur 2010 518 2612 2632 20503430 10.1002/cne.22355

[B28] Janssen R Damen WGM Diverged and conserved aspects of heart formation in a spider Evol Dev 2008 10 155 165 10.1111/j.1525-142X.2008.00223.x 18315809

[B29] Prpic NM Damen WGM Notch-mediated segmentation of the appendages is a molecular phylotypic trait of the arthropods Dev Biol 2009 326 262 271 10.1016/j.ydbio.2008.10.049 19046962

[B30] Anderson DT Embryology and phylogeny in annelids and arthropods 1973 Oxford, New York: Pergamon

[B31] Holm Å Experimentelle Untersuchungen über die Entwicklung und die Entwicklungsphysiologie des Spinnenembryos Zool Bid Uppsala 1952 29 293 424

[B32] Chaw RC Vance E Black SD Gastrulation in the spider *Zygiella x-notata *involves three distinct phases of cell internalization Dev Dyn 2007 236 3484 3495 10.1002/dvdy.21371 17994544

[B33] Yoshikura M Embryological studies on the liphistiid spider, *Heptathela kimurai* - Part II Kumamoto J Sci 1955 2 1 86

[B34] Yoshikura M Embryological studies on the liphistiid spider, *Heptathela kimurai *- Part I Kumamoto J Sci 1954 3 41 48

[B35] Wallstabe P Beiträge zur Kenntnis der Entwicklungsgeschichte der Araneinen. Die Entwicklung der äusseren Form und Segmentierung Zool Jb Abt Anat Ontog Tiere 1908 26 683 712

[B36] Rempel JG The embryology of the black widow spider *Latrodectus mactans *(Fabr.) Can J Zool 1957 35 1 42 10.1139/z57-001

[B37] Liu Y Maas A Waloszek D Early development of the anterior body region of the grey widow spider *Latrodectus geometricus *Koch, 1841 (Theridiidae, Araneae) Arthr Struct Dev 2009 38 401 416 10.1016/j.asd.2009.04.001 19374954

[B38] Weygoldt P Barth FA Ontogeny of the arachnid central nervous system Neurobiology of Arachnids 1985 Berlin: Springer-Verlag 20 37

[B39] Weygoldt P Untersuchungen zur Embryologie und Morphologie der Geilßelspinne *Tarantula marginemaculata *C. L. Koch (Arachnida, Amblypygi, Tarantulidae) Zoomorphologie 1975 82 137 199 10.1007/BF00993587

[B40] Machner J Scholtz G A scanning electron microscopy study of the embryonic development of *Pycnogonum litorale *(Arthropoda, Pycnogonida) J Morph 2010 271 1306 1318 10.1002/jmor.10871 20658562

[B41] Foelix RF Biology of spiders 1996 New York: Oxford University Press

[B42] Purcell WF The phylogeny of the tracheae in Araneae Quart J Microsc Sci 1910 54 519 563

[B43] Kimble M Coursey Y Ahmad N Hinsch G Behavior of the yolk nuclei during embryogenesis, and development of the midgut diverticulum in the Horseshoe Crab *Limulus polyphemus* Invert Biol 2002 121 365 377

[B44] Akiyama-Oda Y Oda H Axis specification in the spider embryo: *dpp *is required for radial-to-axial symmetry transformation and *sog *for ventral patterning Development 2006 133 2347 2357 10.1242/dev.02400 16720876

[B45] Farley RD Development of segments and appendages in embryos of the desert scorpion *Paruroctonus mesaensis *(Scorpiones: Vaejovidae) J Morph 2001 250 70 88 10.1002/jmor.1060 11599017

[B46] Holm Å Studien über die Entwicklung und Entwicklungsbiologie der Spinnen Zool Bid Uppsala 1940 19 1 214

[B47] Downes MF A proposal for standardization of the terms used to desribe the early development of spiders, based on a study of *Theridion rufipes *Lucas (Araneae: Theridiidae) Bull Br ArachnoI Soc 1987 7 187 193

[B48] Stollewerk A Seyfarth EA Evolutionary changes in sensory precursor formation in arthropods: Embryonic development of leg sensilla in the spider *Cupiennius salei* Dev Biol 2008 313 659 673 10.1016/j.ydbio.2007.11.003 18054903

[B49] Manton SM Studies on the Onychophora. VII. The early embryonic stages of *Peripatopsis*, and some general considerations concerning the morphology and phylogeny of the Arthropoda Phil Trans Roy Soc London B 1949 233 483 580 10.1098/rstb.1949.0003

[B50] Dohle W Die Embryonalentwicklung von *Glomeris marginata *(Villers) im Vergleich zur Entwicklung anderer Diplopoden Zool Jb Anat 1964 81 241 310

[B51] Wolff C Scholtz G Cell lineage, axis formation, and the origin of germ layers in the amphipod crustacean *Orchestia cavimana* Dev Biol 2002 250 44 58 10.1006/dbio.2002.0789 12297095

[B52] Machida R Nagashima T Ando H The early embryonic development of the jumping bristletail *Pedetontus unimaculatus *Machida (Hexapoda: Microcoryphia, Machilidae) J Morph 1990 206 181 195 10.1002/jmor.1052060205 29865763

[B53] Kautzsch G Über die Entwicklung von *Agelena labyrinthica *Clerck Zool Jb Abt Anat Ontog Tiere 1909 28 477 538

[B54] Oda H Nishimura O Hirao Y Tarui H Agata K Akiyama-Oda Y Progressive activation of Delta-Notch signaling from around the blastopore is required to set up a functional caudal lobe in the spider *Achaearanea tepidariorum* Development 2007 134 2195 2205 10.1242/dev.004598 17507394

[B55] Yamazaki K Akiyama-Oda Y Oda H Expression patterns of a *twist *related gene in embryos of the spider *Achaearanea tepidariorum *reveal divergent aspects of mesoderm development in the fly and spider Zool Sci 2005 22 177 185 10.2108/zsj.22.177 15738638

[B56] Scholtz G Homology and ontogeny: Pattern and process in comparative developmental biology Theory Biosci 2005 124 121 143 17046352 10.1007/BF02814480

[B57] Stollewerk A Simpson P Evolution of early development of the nervous system: a comparison between arthropods Bioessays 2005 27 874 883 10.1002/bies.20276 16108062

[B58] Dogiel V Embryologische Studien an Pantopoden Z Wiss Zool 1913 57 577 741

[B59] Morgan TH A contribution to the embryology and phylogeny of the pycnogonids Stud Biol Lab, Johns Hopkins Univ 1891 5 1 76

[B60] Scholtz G Edgecombe GD The evolution of arthropod heads: reconciling morphological, developmental and palaeontological evidence Dev Genes Evol 2006 216 395 415 10.1007/s00427-006-0085-4 16816969

[B61] Montgomery TH The development of the *Theridium*, an aranead, up to the stage of reversion J Morph 1909 20 298 351

[B62] Kimm M Prpic NM Formation of the arthropod labrum by fusion of paired and rotated limb-bud-like primordia Zoomorphology 2006 125 147 155 10.1007/s00435-006-0019-8

[B63] Crome W Embryonalentwicklung ohne "Umrollung" (= Reversion) bei Vogelspinnen (Araneae: Orthognatha) Deutsche Entomol Z 1963 10

[B64] Holm Å Notes on the development of an orthognath spider, *Ischnothele karschi *Bös. & Lenz Zool Bid Uppsala 1954 30 199 222

[B65] Damen WGM Evolutionary conservation and divergence of the segmentation process in arthropods Dev Dyn 2007 236 1379 1391 10.1002/dvdy.21157 17440988

[B66] Dunlop JA Selden PA The origin of tetrapulmonate book lungs and their significance for chelicerate phylogeny Proceedings of the 17th European Colloquium of Arachnology 1998 Edinburgh 1997 9 16

[B67] Kassianow N Die Frage über den Ursprung der Arachnoideenlungen aus den Merostomenkiemen (Limulus-Theorie) Biol Centralblatt 1914 34 8 46 108-149, 170-213, 221-247

[B68] Bromhall C Spider tracheal systems Tiss Cell 1987 19 793 807 10.1016/0040-8166(87)90020-6 18620223

[B69] Shultz J A phylogenetic analysis of the arachnid orders based on morphological characters Zool J Linn Soc 2007 150 221 265 10.1111/j.1096-3642.2007.00284.x

[B70] Westheide W Rieger R Spezielle Zoologie Teil 1: Einzeller und Wirbellose Tiere 2006 Spektrum Akademischer Verlag

[B71] Vachon M Contribution à l'étude du développement post-embryonnaire des araignées. Première note. Généralites et nomenclature des stades Bull Soc Zool Fr 1957 82 337 354

[B72] Kleinteich A Schneider JM Developmental strategies in an invasive spider: constraints and plasticity Ecol Entomol 2011 36 82 93 10.1111/j.1365-2311.2010.01249.x

[B73] Schnabel R Hutter H Moerman D Schnabel H Assessing normal embryogenesis in *Caenorhabditis elegans *using a 4D microscope: Variability of development and regional specification Dev Biol 1997 184 234 265 10.1006/dbio.1997.8509 9133433

[B74] Prpic NM Schoppmeier M Damen WGM Collection and fixation of spider embryos Cold Spring Harb Protoc 2008 10.1101/pdb.prot5067 21356698

