# Supplementary material for: The embryonic development of the central American wandering spider Cupiennius salei
Source: Front Zool. 2011 Jun 14;8:15. doi: 10.1186/1742-9994-8-15 (PMC3141654; doi:10.1186/1742-9994-8-15)
Supplement: Additional file 1 — Tabular overview, comparing the new staging system and nomenclature for the development of C. salei with the system and nomenclature used in earlier publications. hAEL: hours after egg laying, *[26] **[7] ***[48]. Names/annotation of the stages used in earlier publications are provided, along with an English translation: Frühe Periplasmafelderung - Early periplasma aggregation; Kerne - nuclei; Späte Furchung - late cleavage; (Großzelliges/Frühes) Blastoderm.- (large-cell/early) blastoderm; (Späte) Eikontraktion - (late) egg contraction; (Frühe/Späte) Primitivplatte (dreischichtig) - (early/late) primitive plate (three-layered); (Früher/ Später) Primitivpfropf - (early/late) primary thickening; Keimhemisphärenausbildung - germ field formation; Keimhemisphäre - germ field; Später unsegmentierter Keimstreif - (late) unsegmented germ band; Thoraxsegmentierung - thorax segmentation; Bildung der Thoraxextremitäten - development of prosomal appendages; Abdominale Segmentierung - opisthosomal segmentation; Abdominalknospenbildung - opisthosomal limb bud formation; Umrollung - inversion; Abgeknickter Embryo - crooked embryo; Streckung des Embryos - stretching of the embryo; Erstes Postembryonalstadium - first post-embryonic stage; (1./2.) Prälarva - (first/second) prelarva; Jungspinne - spiderling; Larve - larva. [file 1742-9994-8-15-S1.PDF]

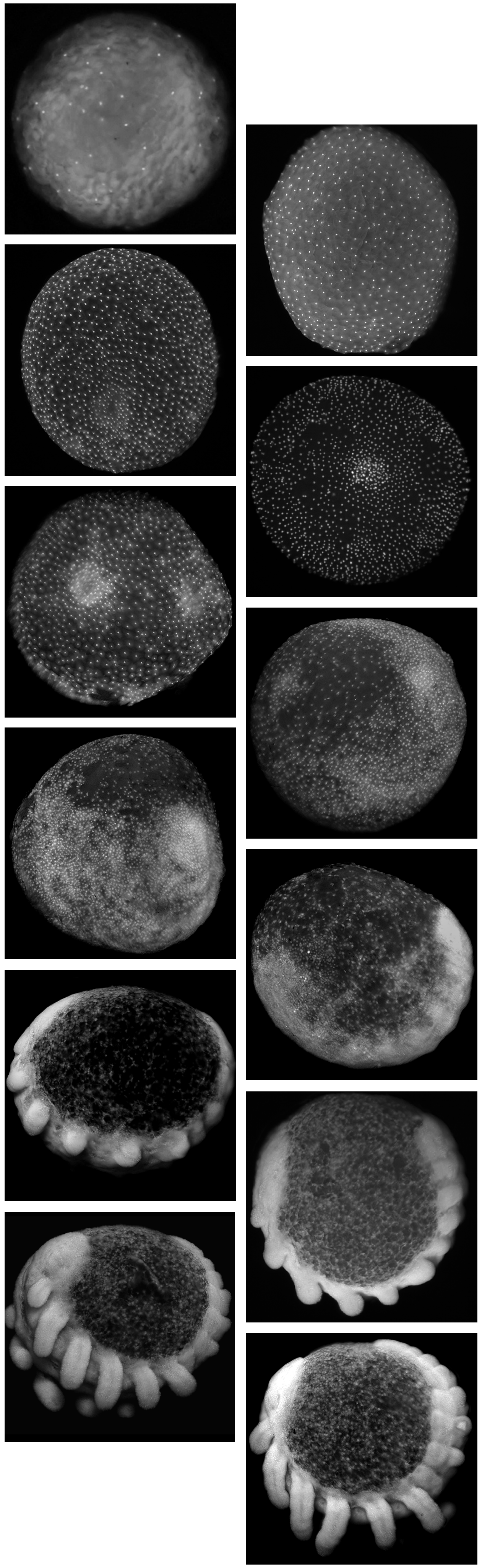

New Staging System		Staging after Seitz* and others**, ***	
Nr.	Name	hAEL	Name
1	Early cleavages	4<br>4-8<br>10-12<br>12-14<br>18-22	*Frühe Periplasmafelderung*<br>*2 Kerne*<br>*4 Kerne*<br>*8 Kerne*<br>*Späte Furchung*
2	Blastoderm	22-26<br>26-30<br>30-35	*Frühes Blastoderm*<br>*Großzelliges Blastoderm*<br>*Blastoderm*
3	Blastopore	35-48<br>48-52	*Eikontraktion*<br>*Späte Eikontraktion*
4	Primary thickening	52<br>55<br>60<br>62<br>65-70<br>72	*Frühe Primitivplatte*<br>*Späte Primitivplatte*<br>*Primitivplatte dreischichtig*<br>*Früher Primitivpfropf*<br>*Primitivpfropf*<br>*Später Primitivpfropf*
5	Cumulus migration	75-80<br>80<br>82	*Keimhemisphärenausbildung*<br>*Keimhemisphäre*<br>*Unsegmentierter Keimstreif*
6	Dorsal field	85	*Unsegmentierter Keimstreif*
7	Germ band	85-100	*Später unsegmentierter Keimstreif bis Thoraxsegmentierung*
8	Segmented germ band	85-100	*Später unsegmentierter Keimstreif bis Thoraxsegmentierung*
9	Prosomal limb buds	100-130	*Bildung der Thoraxextremitäten*
10	Prosomal limb bud elongation	130-160	*Abdominale Segmentierung*
11	Opisthosomal limb buds	160-180	*Abdominalknospenbildung*
12	Lateral furrow		

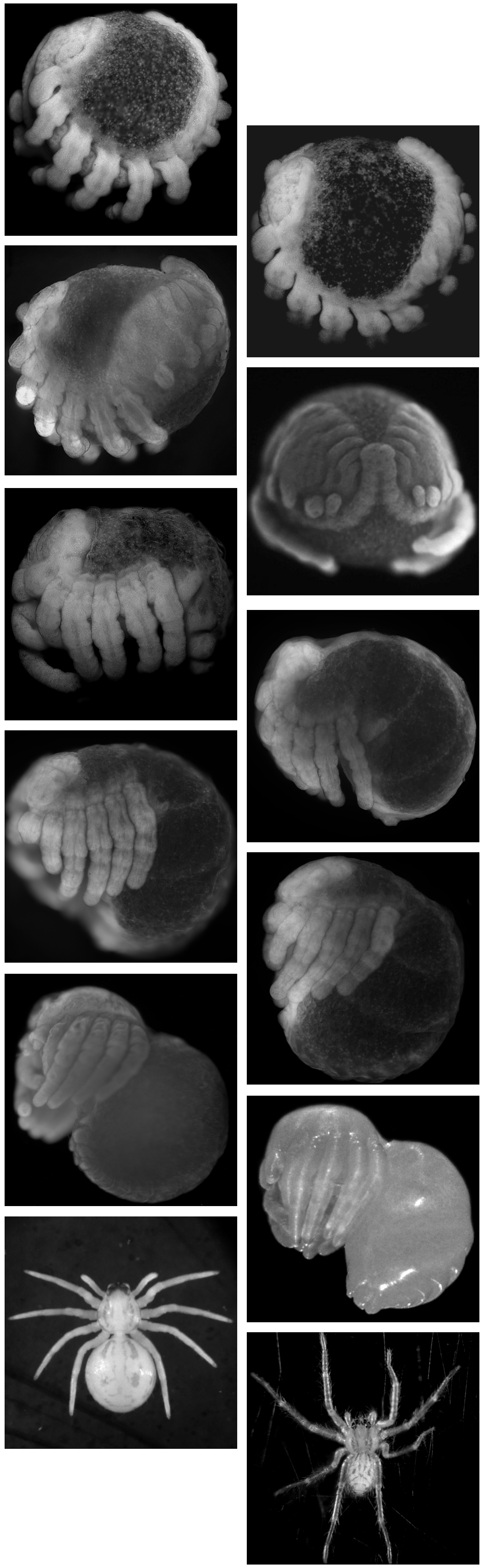

New Staging System		Staging after Seitz* and others**, ***	
Nr.	Name	hAEL	Name
13	Labrum	160-180	*Abdominalknospenbildung*
14	Inversion I		
15	Inversion II		
16	Inversion III	180-220	*Umrollung*
17	Dorsal closure		
18	Prosomal shield		
19	Heart	220-280	*Abgeknickter Embryo*
20	Ventral closure		
21	Petiolus	280-320	*Streckung des Embryos* *<br>*-1. Prälarve* **
	**Postembryo**	320-340	*-Erstes Postembryonal-stadium* *<br>*-2. Prälarve* **<br>-stage 1 larva ***
	**1st Instar**	360-380	*-Jungspinne* *<br>*-Larve* **<br>-stage 2 larva ***
	**2nd Instar**		stage 3 larva ***
