# Supplementary figures and images for: The embryonic development of the central American wandering spider Cupiennius salei
Source: Front Zool. 2011 Jun 14;8:15. doi: 10.1186/1742-9994-8-15 (PMC3141654; doi:10.1186/1742-9994-8-15)

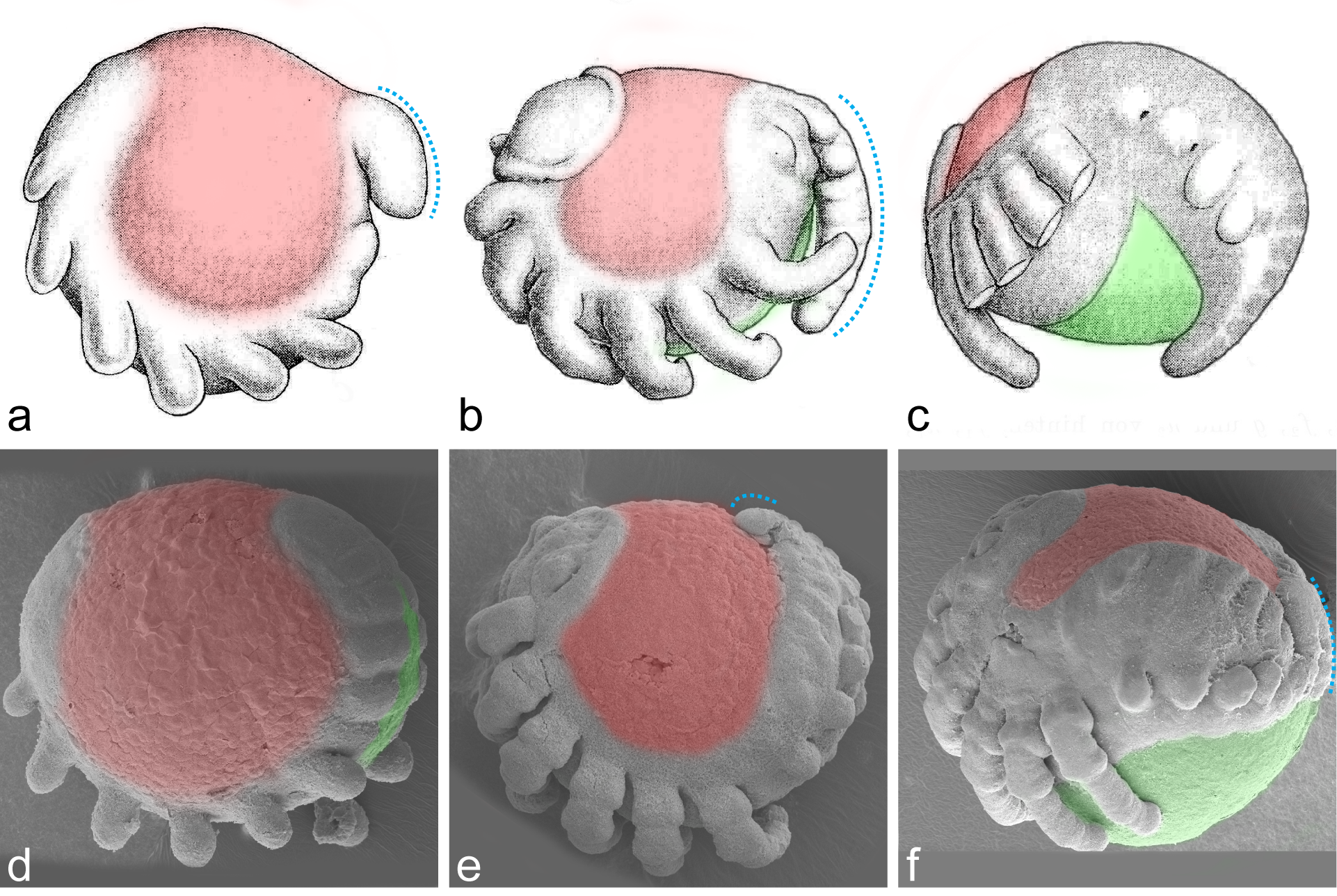

Supplement: Additional file 4 — Comparison of inversion and 'post-opisthosoma' of C. salei and S. bavarica. Drawings modified from [46], a-c; SEMs, d-f. Images are false-coloured to show the exposed area of the yolk mass in red and the ventral sulcus in green. a: Lateral view of an S. bavarica embryo, stage shortly before opisthosomal limb buds appear. The blue dotted line indicates the primordium of the post-opisthosoma. b: Lateral view of an S. bavarica embryo at the onset of inversion. The blue dotted line indicates the ventrally flexed post-opisthosoma, consisting of opisthosomal segments four to twelve. c: Lateral view of an S. bavarica embryo shortly before dorsal closure. d-e: Lateral view of C. salei stages 10 and 14 respectively. Blue dotted line in e indicates the primordium of the post-opisthosoma. f: Dorso-lateral view of C. salei stage 16. Blue dotted line indicates the dorsally flexed post-opisthosoma, consisting of opisthosomal segments nine to twelve. [file 1742-9994-8-15-S4.TIFF]
